# Glucose and Glutamine Deprivation Promotes Breast Cancer Lung Metastasis via BRD4‐Dependent Enhancer Activation

**DOI:** 10.1002/advs.77493

**Published:** 2026-08-29

**Authors:** Poshan Yugal Bhattarai, Arathy Vasukutty, Yujin Na, Sung‐Chul Lim, Hong Seok Choi

**Affiliations:** ^1^ Honam Regional Future Drug Development Convergence Education & Research Division (BK21 FOUR) College of Pharmacy Chosun University Jeonnam‐Gwangju Special Metropolitan City 61452 Republic of Korea; ^2^ Department of Pathology School of Medicine Chosun University Jeonnam‐Gwangju Special Metropolitan City 61452 Republic of Korea

**Keywords:** BRD4, breast cancer metastasis, EDN1, enhancer, enhancer RNA, nutrient deprivation

## Abstract

Nutrient deprivation in the tumor microenvironment drives metastatic progression. However, its role in breast cancer metastasis and underlying epigenetic mechanisms remain unclear. We identify an enhancer‐driven transcriptional program that promotes breast cancer metastasis under nutrient deprivation. Transient glucose–glutamine deprivation in MCF7 and MDA‐MB‐231 cells induces the expression of genes involved in migration, invasion, and metastasis. Chromatin immunoprecipitation sequencing shows distinct enhancer activation, marked by increased histone H3 lysine 27 and bromodomain‐containing protein 4 (BRD4) recruitment. Targeted enhancer acetylation using dCas9–p300 increases cognate gene expression. Motif and CUT&RUN analyses indicate the enrichment and direct binding of ATF3 and c‐JUN at enhancers—essential for their activation. Transient transcriptome sequencing shows an increase in endothelin 1 (*EDN1*) enhancer RNA (eRNA) transcription to facilitate enhancer activation, which is reduced by treatment with eRNA‐targeting antisense oligonucleotides (ASOs). In an orthotopic mouse model, BRD4 inhibition using JQ1 or MZ1 suppresses 4T1 cell metastasis. *EDN1* enhancer acetylation with dCas9–p300 increases MCF7 tumor growth, which is reduced by BRD4 inhibition or ASO treatment. BRD4 and EDN1 expression is commonly upregulated in human breast metastasis. These findings define nutrient stress‐responsive enhancers as epigenetic drivers of metastatic adaptation and highlight their therapeutic potential in breast cancer.

## Graphical abstract

To model a nutrient‐restricted tumor microenvironment, breast cancer cells were adapted to low glucose and glutamine levels. In the adapted cells, BRD4‐driven enhancer activation increases pro‐invasive *EDN1* expression, facilitated by ATF3/c‐JUN and reinforced by enhancer RNA. Disrupting this complex using a BRD4 degrader or antisense oligonucleotides reduces *EDN1* expression and suppresses breast cancer cell invasiveness and metastasis.

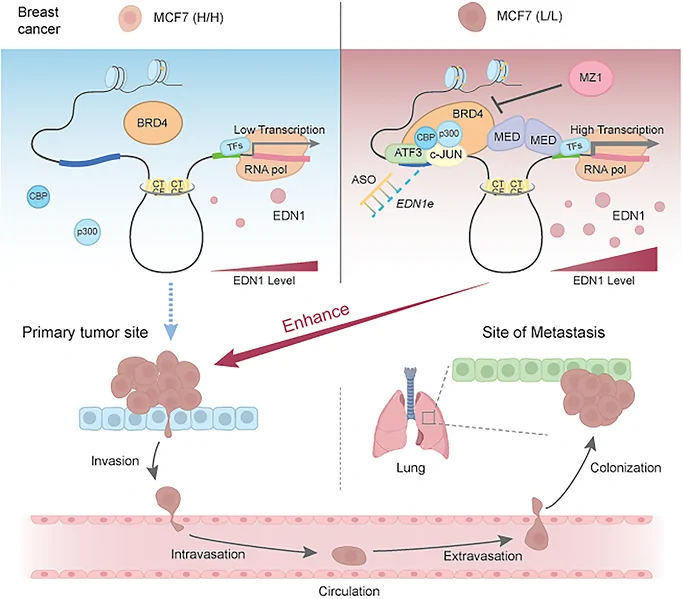

## 1 Introduction

Breast cancer cells prioritize glucose and glutamine as metabolic precursors for central carbon metabolism, making their availability essential for tumorigenesis [1, 2]. Glucose is primarily metabolized via aerobic glycolysis, that is, the Warburg effect, to generate biosynthetic intermediates. To ensure glucose availability, breast cancer cells upregulate glucose transporters, such as GLUT1 [3], and induce systemic hyperglycemia by disrupting insulin secretion [4]. Under glucose limitation, particularly during hypoxia, glutamine uptake and glutaminolysis intensify to sustain tricarboxylic acid cycle anaplerosis and adenosine triphosphate (ATP) production. However, voracious consumption rapidly depletes glutamine levels in the breast tumor core [5]. In multiple cancer models, including those of colon and pancreatic cancers, the depletion of glucose and glutamine triggers adaptive responses that promote cell survival and metastatic behavior [6, 7]. In breast cancer, glucose deprivation induces the migration of cancer cells [8]. However, the adaptation of breast cancer cells to simultaneous glucose and glutamine deprivation and underlying molecular mechanisms remain unclear.

Adaptation to nutrient deprivation is accompanied by extensive transcriptional reprogramming, which enables cancer cell survival under metabolic stress [7]. These transcriptional changes are linked to epigenetic remodeling, which dynamically modulates chromatin accessibility and gene expression [7, 9, 10]. Among key epigenetic regulators, reversible histone acetylation plays a pivotal role in shaping chromatin structure and activating stress‐responsive genes. Acetylation of histone H3 lysine 27 (H3K27ac) is a hallmark of transcriptionally active chromatin [11]. At promoters, H3K27ac marks actively transcribed genes. Meanwhile, at distal regulatory regions, it denotes enhancer and super‐enhancer elements that recruit epigenetic readers, including bromodomain‐containing protein 4 (BRD4), to form an enhancer complex and activate the transcription of target genes. While chromosomal rearrangements commonly activate enhancers, a more rapid and tunable mechanism involves de novo enhancer formation in response to environmental cues such as nutrient deprivation [12]. Notably, BRD4 binding is critical for enhancing enhancer function [13]. BRD4‐bound enhancers are increasingly recognized as key drivers of oncogenic transcriptional programs that contribute to therapeutic resistance, stress adaptation, and metastatic progression [14, 15, 16]. In breast cancer, BRD4 promotes metastatic dissemination and malignancy by activating the Jagged/Notch1 signaling pathway and regulating Snail expression [17, 18]. Nevertheless, the role of BRD4‐mediated enhancer activation in cell survival during nutrient deprivation remains poorly understood. Elucidating how the H3K27ac–BRD4 axis orchestrates transcriptional reprogramming under metabolic stress may provide critical insights into the epigenetic basis of tumor plasticity and survival.

Enhancer activity depends on the coordinated recruitment of transcription factors (TFs) and co‐activators, including p300/CREB‐binding protein and RNA polymerase II [19]. TFs have been studied primarily for their role in promoter activation. However, recent genomic studies showed that most (≈77.5%) TF binding occurs at enhancers rather than promoters, where they orchestrate cell type‐specific transcriptional programs [20]. For instance, 90% of estrogen receptor binding sites are located 10 kb away from the nearest promoter [21]. Identifying nutrient‐deprivation‐induced TFs that bind and activate these dynamic enhancers may enable the direct mapping of stress‐adaptive transcriptional landscapes, elucidation of metastatic reprogramming regulators, and development of targeted therapies that disrupt cellular adaptation.

TFs and BRD4 recruit critical components of the transcriptional machinery, including the positive transcription elongation factor b (composed of CDK9 and cyclin T1) and RNA polymerase II, to activate enhancers [19]. Although they function primarily as drivers of target gene expression through the formation of chromatin loops that spatially juxtapose enhancers with promoters, these interactions facilitate direct transcription from enhancers, generating short bidirectional non‐coding transcripts called enhancer RNA (eRNA) [22]. Once dismissed as transcriptional noise, eRNAs have been recognized as functional regulators [19]. They stabilize enhancer–promoter looping, recruit co‐activators, and enable rapid gene induction [22]. An analysis of >10 000 RNA sequencing (RNA‐seq) samples across 32 cancer types in The Cancer Genome Atlas identified >300 000 precise eRNA loci. The massive scale (>300 000 eRNAs vs ≈20 000 protein‐coding genes) indicates that enhancers are the dominant regulatory architecture in cancer [23]. eRNAs strongly predict patient survival and anticancer drug response [24, 25]. eRNA expression responds dynamically to environmental cues. Hypoxia‐inducible eRNA activates SMG1 under oxygen deprivation to protect heart tissue [26]. However, whether nutrient deprivation triggers eRNA production in stress‐activated enhancers and whether these eRNAs drive metastatic gene expression in breast cancer remain unknown. Since eRNAs are enhancer‐specific and targetable by antisense oligonucleotides (ASOs), identifying nutrient‐responsive eRNAs could uncover therapeutic vulnerabilities beyond broad transcriptional inhibition.

Here, we investigated how limited glucose and glutamine availability reprograms the enhancer landscape in breast cancer cells, identifying a previously unrecognized role of nutrient‐responsive enhancers and eRNAs in driving invasion and metastasis. Through integrated epigenomic–transcriptomic analysis, we identified a subset of enhancers that become selectively activated under nutrient‐restricted conditions, strongly induce eRNA transcription, and drive increased expression of metastasis‐ and invasion‐associated target genes. We further delineated the key TFs and co‐activators responsible for enhancer activation and demonstrated distinct therapeutic strategies targeting enhancer activity and eRNA expression using proteolysis‐targeting chimeras (PROTACs) and ASOs. Our work uncovered epigenetic mechanisms that mediate nutrient‐deprivation‐driven breast cancer metastasis and identified targeted therapeutic approaches with translational potential for inhibiting these metastatic processes.

## 2 Results

### 2.1 Nutrient Deprivation Induced by Glucose and Glutamine Restriction Primes Breast Cancer Cells for Increased Proliferative Capacity and Activates Pro‐Invasive Gene Transcription

**FIGURE 1:**
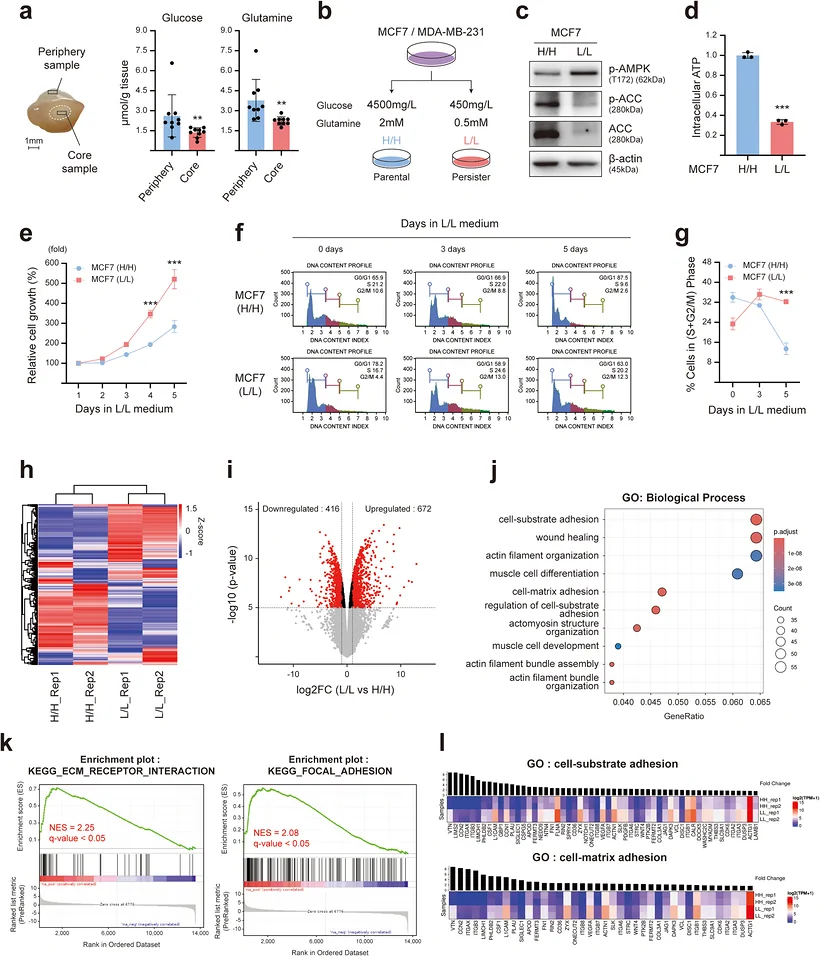
Effect of nutrient deprivation on proliferative and pro‐invasive gene regulation in breast cancer cells. a) Schematic representation of tissue sample collection from tumor core and peripheral regions of MCF7 xenograft tumor (left), and absolute concentrations of glucose and glutamine measured using LC–MS (right). Data are presented as mean ± standard deviation (SD) (*n* = 9). **p < 0.005 (Wilcoxon signed‐rank test). b) Establishment of glucose‐ and glutamine‐deprived MCF7 and MDA‐MB‐231 (L/L) cells from parental (H/H) cells. Commercial GlutaMAX Supplement was used as the glutamine source at the indicated concentrations. c) Western blot analysis of MCF7 (H/H) and MCF7 (L/L) cells probed with the indicated antibodies. d) Intracellular ATP levels in MCF7 (H/H) and MCF7 (L/L) cells measured using an enzyme‐linked immunosorbent assay‐based kit. Data are presented as mean ± SD (*n* = 3). ***p < 0.001 (Student's *t*‐test; compared with H/H). e) Relative proliferation of MCF7 (H/H) and MCF7 (L/L) cells assessed using 3‐(4,5‐dimethylthiazol‐2‐yl)‐2,5‐diphenyltetrazolium bromide assay. Data are presented as mean ± SD (*n* = 3). ***p < 0.001 (Student's *t*‐test; compared with the corresponding H/H group at each time point). f, g) Histogram of cell cycle phases (f) and percentage of MCF7 (H/H) and MCF7 (L/L) cells entering the S and G2/M phases (g). Data are presented as mean ± SD (*n* = 3). ***p < 0.001 (Student's *t*‐test; compared with the corresponding H/H group at day 5). h) Heatmap of differentially expressed genes in MCF7 (H/H) and MCF7 (L/L) cells. The color scale represents Z‐score, with red and blue indicating genes that are upregulated and downregulated, respectively, in MCF7 (L/L) cells relative to MCF7 (H/H) cells. i) Volcano plot showing differentially expressed genes in MCF7 (L/L) cells compared with those in MCF7 (H/H) cells. The number of significant genes [*p* < 0.00001 and |log_2_ fold change (FC)| > 2] is indicated within the figure and highlighted in red. j) Dot plot of top 10 enriched GO biological process terms for genes upregulated in MCF7 (L/L) cells (log_2_FC > 2, false discovery rate < 0.05). Dots are sized by gene count and colored by adjusted *p*‐value (−log_10_ scale). k) Gene set enrichment analysis plot for Kyoto Encyclopedia of Genes and Genomes terms. The normalized enrichment score (NES) and *q*‐value are indicated within each figure. l) Heatmap depicting the expression of individual genes associated with the indicated GO terms. Genes are ordered from left to right based on relative fold change in MCF7 (L/L) versus MCF7 (H/H) cells. The color scale represents log_2_(transcripts per million + 1).

Glucose and glutamine are major metabolic and nutrient substrates for breast cancer cells, yet their intratumoral distribution remains poorly defined. To quantify their intratumoral availability, we isolated tissue samples from the peripheral and core regions of MCF7 xenograft tumors and measured the absolute concentrations of glucose and glutamine using liquid chromatography–mass spectrometry (LC–MS) (Figure 1a, left). Both glucose and glutamine levels were significantly reduced in the tumor core compared with those in the periphery, indicating nutrient deprivation in the tumor core (Figure 1a, right). These findings are consistent with previous reports demonstrating depletion of glutamine and glutamine‐derived amino acids in the tumor core (Figure S1a). To investigate how breast cancer cells adapt to nutrient deprivation, we generated MCF7 (L/L) and MDA‐MB‐231 (L/L) cells, representing distinct clinical characteristics, adapted to low glucose (450 mg/L) and glutamine (0.5 mM) culture medium and compared them with MCF7 (H/H) and MDA‐MB‐231 (H/H) cells maintained under standard high glucose (4500 mg L^−1^) and glutamine (2 mm) conditions (Figure 1b and Figure S1b). To validate the biochemical changes in L/L cells, we assessed the expression of key proteins that sense nutrient deprivation. Immunoblotting showed increased phosphorylated 5′ adenosine monophosphate‐activated protein kinase (AMPK) (Thr172) levels in both MCF7 (L/L) and MDA‐MB‐231 (L/L) cells, suggesting energy stress (Figure 1c and Figure S1c). AMPK activation inhibits acetyl‐CoA carboxylase 1 and 2 (ACC1/2) via Ser79 or 212 phosphorylation to suppress de novo lipogenesis. However, we observed concomitant decreases in both phosphorylated ACC (Ser79) and total ACC levels in MCF7 (L/L) and MDA‐MB‐231 (L/L) cells, consistent with prior reports that glucose availability may regulate ACC levels by directly affecting its transcription [27] (Figure 1c and Figure S1c). Since AMPK is primarily activated by low cellular ATP/AMP ratios, we quantified intracellular ATP levels using enzyme‐linked immunosorbent assay. These were significantly reduced (≈4‐fold) in MCF7 (L/L) cells compared with that in MCF7 (H/H) cells, consistent with the observed AMPK activation (Figure 1d). Breast cancer cells, including MCF7 cells, depend on glucose and glutamine for survival and proliferation. Accordingly, the viability of MCF7 cells was reduced during the 4 d adaptation period in L/L media (Figure S1d). However, upon transfer to fresh L/L medium, the surviving MCF7 (L/L) cells showed enhanced growth compared with that of MCF7 (H/H) cells, as evidenced by MTT assay (Figure 1e). Since MTT absorbance depends on mitochondrial NAD(P)H‐dependent oxidoreductase activity, which may be altered when cultured in L/L media, we assessed proliferation through BrdU incorporation. Compared with MCF7 (H/H) cells, MCF7 (L/L) cells showed increased BrdU incorporation, indicating enhanced proliferation when cultured in L/L media (Figure S1e). Cell cycle analysis showed that MCF7 (L/L) cells successfully advanced from the S phase into the G2/M phase, whereas MCF7 (H/H) cells displayed pronounced G0/G1 arrest (Figure 1f,g). To assess the transcriptional changes supporting the enhanced survival of MCF7 (L/L) cells under nutrient deprivation, we performed RNA‐seq. Although RNA‐seq was performed with a limited sample size (*n* = 2), the reproducibility and suitability of the data for downstream analysis were supported by consistent sample clustering in principal component analysis and appropriate dispersion estimates (Figure S1f,g). Differential gene expression analysis revealed widespread transcriptional changes, with 672 upregulated and 416 downregulated genes in MCF7 (L/L) versus MCF7 (H/H) cells (Figure 1h,i). Gene Ontology (GO) analysis of upregulated genes in MCF7 (L/L) cells revealed significant enrichment in biological processes related to cell adhesion, wound healing, and actin filament organization. These terms are primarily associated with cell movement and migration, suggesting a transcriptional shift toward a more adhesive and migratory phenotype in MCF7 (L/L) cells (Figure 1j). Cell migration activation typically requires coordinated overexpression across multiple genes in related pathways rather than isolated changes in individual genes. Kyoto Encyclopedia of Genes and Genomes pathway enrichment analysis, which provides a robust indication of pathway activation, identified extracellular matrix (ECM)–receptor interaction and focal adhesion as among the most significantly enriched pathways in MCF7 (L/L) cells, both of which promote cell migration, survival, and proliferation (Figure 1k and Figure S1h). To identify specific upregulated genes in MCF7 (L/L) cells contributing to the migratory phenotype, we analyzed differential expression within cell–substrate adhesion and cell–matrix adhesion categories. This revealed several candidates, including vitronectin (VTN) and integrin‐binding proteins (Figure 1l). Collectively, these data identify specific genes and gene sets related to cell migration and proliferation that are upregulated in nutrient‐deprived MCF7 (L/L) cells.

### 2.2 H3K27ac Gain at Putative Enhancer Elements Promotes Invasion‐Associated Gene Expression in Nutrient‐Starved Cells in a BRD4‐Dependent Manner

**FIGURE 2:**
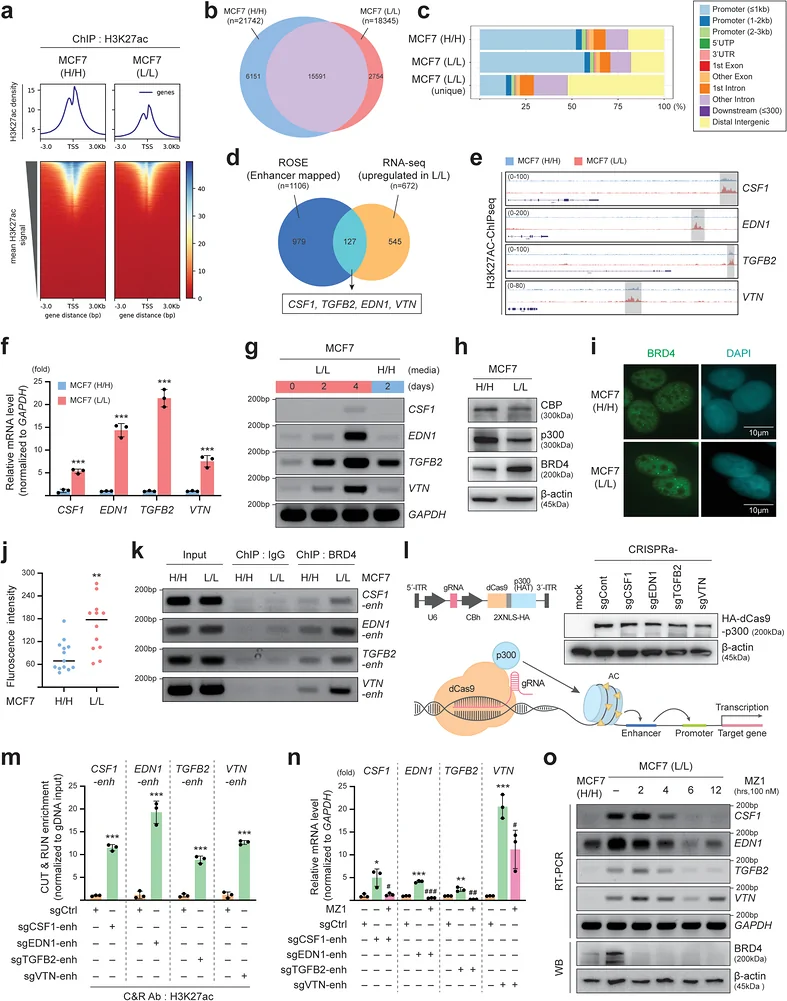
Regulation of H3K27ac‐marked enhancer elements by BRD4 in nutrient‐starved cells. a) H3K27ac ChIP‐seq signal centered on the TSS (upper panel) along with a heatmap displaying peak enrichment [log_2_(ChIP/Input)] around the TSS (lower panel) in MCF7 (H/H) and MCF7 (L/L) cells. b) Venn diagram showing overlap of H3K27ac peaks between MCF7 (H/H) and MCF7 (L/L) cells identified using MACS2. c) Genome‑wide distribution of H3K27ac peaks annotated by genomic feature (left), and color‑coded legend indicating the different genomic regions (right). d) Venn diagram showing the overlap between genes upregulated in MCF7 (L/L) cells and genes mapped to nearby enhancers using the genemapper function in ROSE. e) Integrative Genomics Viewer plot of H3K27ac signals at putative enhancers of indicated genes in MCF7 (H/H) and MCF7 (L/L) cells. The putative enhancer region is highlighted in grey. f) RT‐qPCR with primers targeting indicated genes in MCF7 (H/H) and MCF7 (L/L) cells. Data are presented as mean ± SD (*n* = 3). ***p < 0.001 (Student's *t*‐test; compared with H/H). g) RT‐PCR using primers targeting the indicated genes in MCF7 cells cultured in L/L medium for the indicated days, followed by recovery in H/H medium for 2 d. h) Western blot analysis for indicated enhancer‐associated proteins in MCF7 (H/H) and MCF7 (L/L) cells. i, j) Immunofluorescence staining of MCF7 (H/H) and MCF7 (L/L) cells using anti‐BRD4 antibody (green) and 4′,6‐diamidino‐2‐phenylindole to detect nuclear DNA (blue) (i); quantification of fluorescence intensity of BRD4 foci (j). Data are presented as mean ± SD (*n* = 3). **p < 0.005 (Student's *t*‐test; compared with H/H). k) BRD4 ChIP‐PCR with primers targeting indicated enhancer region (denoted by the “‑enh” suffix) in MCF7 (H/H) and L/L cells. l) Schematic map of CRISPRa construct encoding dCas9–p300 fusion (top, left) and expression of dCas9–p300 protein in HEK293 cells detected using an anti‑HA tag antibody (top, right). The CRISPRa mechanism involves dCas9–p300‐mediated H3K27ac deposition at specific enhancer loci specified by guide RNAs, leading to activation of target genes (bottom). m) CUT&RUN–qPCR showing H3K27ac enrichment at the indicated enhancer regions in HEK293 cells transfected with CRISPRa plasmids expressing sgCtrl, sgCSF1‐enh, sgEDN1‑enh, sgTGFB2‑enh, and sgVTN‑enh. Data are presented as mean ± SD (*n* = 3). ***p < 0.001 (Student's *t*‐test; compared with sgCtrl). n) RT‐qPCR of indicated genes in HEK293 cells transfected with CRISPRa plasmids expressing sgCtrl, sgCSF1‐enh, sgEDN1‐enh, sgTGFB2‐enh, or sgVTN‐enh, followed by treatment with MZ1 (100 nm) for 24 h. Data are presented as mean ± SD (*n* = 3). ***^, ###^ *p* < 0.001, **^, ##^ *p* < 0.05, *^, #^ *p* < 0.05 [one‐way analysis of variance (ANOVA); *,**,*** compared with sgCtrl; #, ##, ### compared with sgEnhancer/MZ1(‐)] o) RT‐PCR of MCF7 (H/H) and MCF7 (L/L) cells treated with MZ1 for the indicated durations using primers targeting the indicated genes and detection of protein levels using western blotting in cell lysates under the corresponding conditions with the indicated antibodies.

Transcriptional regulation is the primary mechanism controlling mRNA levels in cells under specific environmental conditions [28]. To map the active regulatory landscape (enhancers and promoters) that regulates gene transcription in MCF7 (L/L) cells, we performed H3K27ac chromatin immunoprecipitation sequencing (ChIP‐seq). H3K27ac peaks were significantly enriched around the transcription start site (TSS) in both MCF7 (H/H) and MCF7 (L/L) cells, with MCF7 (L/L) cells showing reduced peak intensity, suggesting globally attenuated transcription (Figure 2a and Figure S2a). A total of 21,742 and 18,345 H3K27ac peaks with *q* < 0.05 were identified in MCF7 (H/H) and MCF7 (L/L) cells, respectively. Of these, 6,151 and 2,754 peaks were unique to MCF7 (H/H) and MCF7 (L/L) cells, respectively (Figure 2b).

To assess the functional significance of H3K27ac in gene expression, we grouped H3K27ac peaks into three categories, MCF7 (H/H), MCF7 (L/L), and MCF7 (L/L)‐unique, as described in the Supplementary Materials and Methods, and analyzed their genome‐wide distribution (Figure 2c). In both the MCF7 (H/H) and MCF7 (L/L) groups, >60% of H3K27ac peaks were located within promoter regions (defined as ± 3 kb of the TSS), suggesting that H3K27ac modification is mostly associated with the promoter function. By contrast, >75% of MCF7 (L/L)‐unique H3K27ac peaks were enriched in distal intergenic and intronic regions (Figure 2c). H3K27ac modification at these regions may represent enhancer activation. Collectively, despite a global reduction in promoter‑associated H3K27ac levels in MCF7 (L/L) cells, we observed differential H3K27ac at a subset of distal regions consistent with enhancer activation, which may underlie the selective upregulation of specific genes in MCF7 (L/L) cells.

To more precisely identify enhancer elements, we analyzed H3K27ac peaks using the rank ordering of super‐enhancers (ROSE) algorithm, which identifies and ranks both typical enhancers and super‐enhancers (Figure S2b). Because we are interested in enhancers that are exclusively present in MCF7 (L/L) cells, we identified enhancers from MCF7 (L/L)‐unique H3K27ac peaks and mapped them to nearby genes (Figure 2d and Figure S2c). We found several MCF7 (L/L)‐unique enhancers adjacent to genes overexpressed in MCF7 (L/L) cells. For subsequent analyses, we selected representative candidates including colony‐stimulating factor 1 (*CSF1*), endothelin 1 (*EDN1*), transforming growth factor beta 2 (*TGFB2*), and VTN based on their known association with cancer cell invasion and metastasis (Figure 2e and Figure S2c).

To validate these findings, we performed reverse transcription‐quantitative polymerase chain reaction (RT‑qPCR) to assess *CSF1*, *EDN1*, *TGFB2*, and *VTN* expression, which were significantly upregulated in MCF7 (L/L) cells compared with that in MCF7 (H/H) cells (Figure 2f). Consistently, EDN1 expression was significantly increased in MDA‐MB‐231 (L/L) cells compared with that in MDA‐MB‐231 (H/H) cells (Figure S2d), indicating a partially distinct expression pattern compared with that observed in MCF7 cells. Time‑course experiments in which MCF7 cells were cultured in L/L medium and then recovered in H/H medium revealed conditional activation of *CSF1*, *TGFB2*, *EDN1*, and *VTN* depending on nutrient availability (Figure 2g).

Since H3K27ac at enhancers is primarily catalyzed by the histone acetyltransferases p300/CBP, which in turn recruit bromodomain‑containing proteins such as BRD4 to facilitate assembly of a functional enhancer complex, we examined p300, CBP, and BRD4 protein levels in MCF7 (L/L) and MDA‐MB‐231 (L/L) cells. CBP and p300 protein levels were reduced, whereas BRD4 expression modestly increased in both L/L cell lines (Figure 2h and Figure S2e). Overexpression of BRD4 resulted in increased formation of BRD4 nuclear foci in MCF7 (L/L) cells compared with that in MCF7 (H/H) cells (Figure 2i,j). Although the exact molecular composition of BRD4 nuclear foci is not fully defined, they are typically associated with super‑enhancer complexes and increase under stress conditions, including heat shock and viral infection [29]. To validate BRD4 recruitment to enhancer elements, we performed ChIP‐PCR/qPCR using a BRD4 antibody, which revealed significantly increased BRD4 occupancy at the putative enhancers of *CSF1*, *EDN1*, *TGFB2*, and *VTN* in MCF7 (L/L) cells compared with that in MCF7 (H/H) cells (Figure 2k and Figure S2f). Since BRD4 recruitment is preceded by CBP/p300‐mediated H3K27ac, we performed CUT&RUN–qPCR and found that p300 occupancy at these enhancers increased despite a global reduction in p300 expression (Figure S2g). Collectively, these data show that overexpression of *CSF1*, *TGFB2*, *EDN1*, and *VTN* in MCF7 (L/L) cells is accompanied by BRD4 overexpression and its recruitment to putative enhancer elements of these genes.

To directly test whether the enhancer elements discovered in the ROSE analysis are involved in the transcriptional upregulation of *CSF1*, *EDN1*, *TGFB2*, and *VTN* in MCF7 (L/L) cells, we generated CRISPR activation (CRISPRa) constructs using a dCas9–p300 fusion that enables specific acetylation of enhancer loci guided by sequence‑specific guide RNAs (Figure 2l, left). CRISPRa plasmids encoding guide RNAs targeting the enhancer regions of *CSF1*, *EDN1*, *TGFB2*, and *VTN*, along with a control guide RNA, were introduced into HEK293 cells (Figure 2l, top right). CUT&RUN analysis demonstrated that, compared with transfection with control guide RNA, the CRISPRa system significantly increased H3K27ac levels at the enhancer regions of all the genes (Figure 2m). This CRISPRa‑mediated H3K27ac modification at enhancer regions increased the expression of their corresponding target genes, an effect attenuated by treatment with the BRD4‑targeting PROTAC degrader MZ1 (Figure 2n). Together, these data link the enhancers to their cognate genes and show that increasing H3K27ac at enhancers can directly activate *CSF1*, *TGFB2*, *EDN1*, and *VTN* expression in a BRD4‑dependent manner. To further validate our findings, we selected *EDN1* as a representative candidate and generated a MCF7 monoclonal cell line harboring a CRISPR/Cas9‐mediated deletion of the *EDN1* enhancer region (Figure S2h,i). Enhancer deletion markedly attenuated nutrient deprivation‐induced *EDN1* mRNA expression, demonstrating that *EDN1* expression under nutrient deprivation is enhancer‐dependent (Figure S2j). Finally, we examined whether BRD4 is essential for the increased gene expression under nutrient‑deprived conditions. Treatment with MZ1 significantly reduced *CSF1*, *EDN1*, *TGFB2*, and *VTN* expression in MCF7 (L/L) cells in a time‑dependent manner compared with that in MCF7 (H/H) cells, indicating that BRD4 is required for enhancer‑dependent transcriptional activation of these genes in MCF7 (L/L) cells (Figure 2o; Figure S2k,l).

### 2.3 Cooperative Binding and Co‐Occupancy of JUN and Activating Transcription Factor 3 (ATF3) with BRD4 at Enhancer Elements Activate Starvation‐Responsive Gene Transcription

**FIGURE 3:**
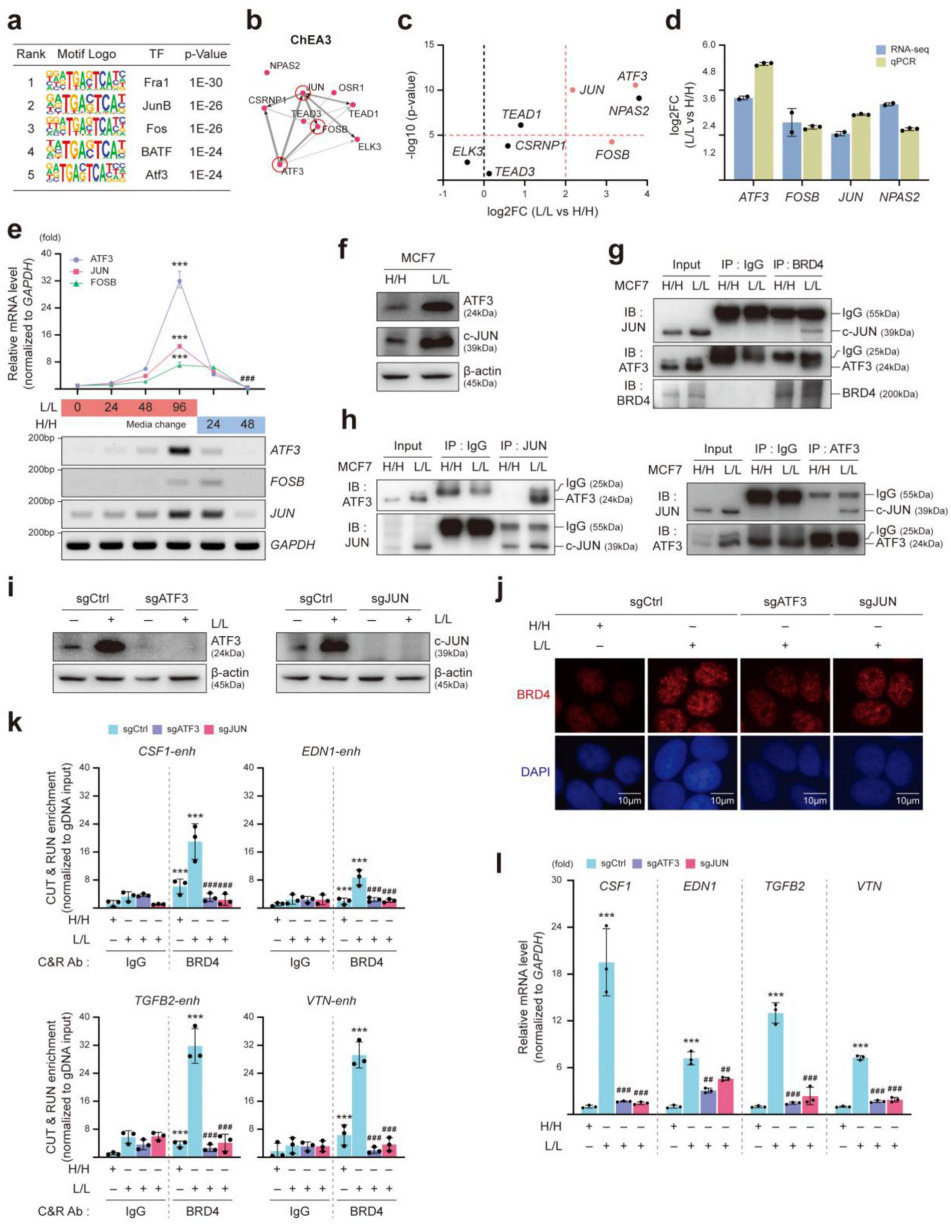
JUN–ATF3–BRD4 cooperative interactions at enhancer elements in starved cells. a) TF motif logo showing enrichment in MCF7 (L/L)‐unique enhancer regions identified using HOMER with a known TF database. The *p*‐value for each motif is shown. b) TF coregulatory network based on ChEA3 analysis of genes overexpressed in MCF7 (L/L) cells. The increased thickness of the edge represents a higher weight and greater significance. c) Volcano plot showing differential expression of ChEA3‐discovered TFs in MCF7 (L/L) cells compared with that in MCF7 (H/H) cells based on RNA‐seq. d) RT‐qPCR validation of differentially expressed TFs in MCF7 (L/L) versus MCF7 (H/H) cells, with corresponding RNA‐seq‐based expression changes. e) RT‑qPCR and RT‑PCR of indicated genes in MCF7 cells cultured in L/L medium for the specified durations, followed by recovery in H/H medium for the indicated durations. Data are presented as mean ± SD (*n* = 3). ***^, ###^ *p* < 0.001 (Student's *t*‐test; *** compared with 0 h; ### compared with 96 h). f) Western blot analysis of MCF (H/H) and MCF7 (L/L) cells using antibodies targeting the indicated proteins. g) Immunoprecipitation of lysate from MCF7 (H/H) and MCF7 (L/L) cells with anti‐BRD4 antibody followed by immunoblotting with indicated antibodies. h) Immunoprecipitation of lysates from MCF7 (H/H) and MCF7 (L/L) cells with anti‐c‐JUN (left) and anti‐ATF3 (right) antibodies followed by immunoblotting with the indicated antibodies. i) Western blot analysis of MCF7 wild‐type cells transfected with sgCtrl and gene‐knockout cells transfected with sgATF3 (left) or sgJUN (right) after 4 d of culture in H/H or L/L medium, probed with the indicated antibodies. j) Immunofluorescence staining for BRD4 foci (red) in MCF7 wild‐type (sgCtrl) and gene‐knockout (sgATF3 and sgJUN) cells cultured in H/H or L/L medium for 4 d. Cell nuclei are counterstained with 4′,6‐diamidino‐2‐phenylindole (blue). Scale bar = 10 µm. k) CUT&RUN–qPCR showing BRD4 enrichment at indicated enhancer regions in MCF7 wild‐type (sgCtrl) and gene‐knockout (sgATF3 and sgJUN) cells cultured in H/H or L/L medium for 4 d. Data are presented as mean ± SD (*n* = 3). ***, ^###^ *p* < 0.001 (Student's *t*‐test; ***compared with H/H‐BRD4 group; #### compared with L/L‐BRD4 group). l) RT‐qPCR of MCF7 wild‐type (sgCtrl) and gene‐knockout (sgATF3 and sgJUN) cells cultured in H/H or L/L medium for 4 d with primers targeting indicated genes. Data are presented as mean ± SD (*n* = 3). ***^, ###^ *p* < 0.001, ^##^ *p* < 0.005 (Student's *t*‐test; *** compared with H/H‐sgCtrl group; ##, ### compared with L/L‐sgCtrl group).

Binding of TFs to enhancer sequences is a prerequisite for enhancer complex assembly and activation. To identify candidate TFs that bind to MCF7 (L/L)‐unique enhancers, we searched for overrepresented motifs of known TFs using the HOMER tool. In total, we identified 48 motifs with *p* < 0.01. Analysis of the most significant motifs revealed enrichment of binding sites for TFs such as Fra1, JUN, FOS, BATF, and ATF3 within MCF7 (L/L)‐unique enhancers (Figure 3a). These TFs belong to the AP‑1 family, consistent with the established role of AP‑1 signaling in stress responses and enhancer activation [30].

As an orthogonal approach, we used ChEA3 to identify TFs that may regulate gene expression in MCF7 (L/L) cells. ChEA3 analyzes user‑defined gene sets—in this case, genes upregulated in MCF7 (L/L) cells—and ranks TFs predicted to regulate these genes based on public ChIP‑seq and RNA‑seq datasets. This analysis highlighted a TF network in which JUN, FOSB, and ATF3 emerged as top candidates for controlling gene expression in MCF7 (L/L) cells (Figure 3b).

Because both HOMER and ChEA3 provide predictive results, we considered the engagement of these TFs under our experimental conditions. The RNA‑seq data revealed that *ATF3*, *JUN*, *NPAS2*, and *FOSB* expression was significantly increased in MCF7 (L/L) cells compared with that in MCF7 (H/H) cells (Figure 3c). qPCR validated these findings (Figure 3d).

Given the reported roles of AP‐1 family TFs including ATF3, JUN, and FOSB in stress adaptation, we examined whether their expression is regulated by nutrient availability. Time‐course analysis showed that *ATF3*, *FOSB*, and *JUN* expression increased during culture in L/L medium and was reversed upon switching to H/H medium, suggesting that activation of these TFs is conditional and occurs specifically under low‑nutrient conditions (Figure 3e). Consistent with MCF7 (L/L) cells, *ATF3*, *FOSB*, and *JUN* expression was significantly increased in MDA‐MB‐231 (L/L) cells compared with that in MDA‐MB‐231 (H/H) cells (Figure S3a). Because *ATF3* and *JUN* showed the largest increase in mRNA expression in MCF7 (L/L) cells and are known to form transcriptionally active dimers, we selected these candidate TFs for further analysis. To determine whether increased mRNA levels translate into protein expression, we performed immunoblotting and confirmed elevated ATF3 and c‑JUN expression in both MCF7 (L/L) and MDA‐MB‐231 (L/L) cells compared with that in MCF7 (H/H) and MDA‐MB‐231 (H/H) cells (Figure 3f and Figure S3b). Together, these data identify ATF3 and JUN as strong candidates that may bind to specific enhancers activated by nutrient deprivation in MCF7 (L/L) and MDA‐MB‐231 (L/L) cells.

To investigate whether ATF3 and c‐JUN recruit BRD4 to enhancers, we first examined their binding to enhancer sequences. CUT&RUN assays showed that both ATF3 and c‐JUN bind to enhancers for *CSF1*, *EDN1*, *TGFB2*, and *VTN*, with increased binding in MCF7 (L/L) cells (Figure S3c,d). We examined the physical interaction between BRD4 and ATF3/c‐JUN using co‐immunoprecipitation and proximity ligation assay (PLA) and observed increased binding of BRD4 to both ATF3 and c‐JUN in MCF7 (L/L) cells compared with that in MCF7 (H/H) cells (Figure 3g and Figure S3e). In addition, dimer formation between ATF3 and c‐JUN was higher in MCF7 (L/L) cells than in MCF7 (H/H) cells as revealed by co‐immunoprecipitation (Figure 3h). Moreover, these interactions were observed exclusively in the nucleus, consistent with their roles in enhancer binding and activation (Figure S3e). Together, these data suggest that ATF3 and c‐JUN dimerization may participate in the enhancer recruitment of BRD4 in MCF7 (L/L) and MDA‐MB‐231 (L/L) cells.

To directly test the role of ATF3 and c‐JUN in enhancer activity, we knocked out both genes separately in MCF7 cells using CRISPR/Cas9 and confirmed that their expression was abolished under L/L culture conditions (Figure 3i). Immunofluorescence staining showed that the increased BRD4 foci formation observed in MCF7 (L/L) cells was markedly reduced upon knockout of either *ATF3* or *JUN*, suggesting these genes participate in BRD4 recruitment (Figure 3j). We directly assessed BRD4 recruitment to specific enhancers using CUT&RUN–qPCR and found that loss of *ATF3* and *JUN* reduced BRD4 occupancy at the enhancers of *CSF1*, *EDN1*, *TGFB2*, and *VTN* (Figure 3k). Consistent with reduced BRD4 binding to the enhancer sequence, the elevated expression of *CSF1*, *EDN1*, *TGFB2*, and *VTN* in MCF7 (L/L) cells was reduced upon knockout of either *ATF3* or *JUN* (Figure 3l). Collectively, these data demonstrate that ATF3 and c‑JUN TFs bind and recruit BRD4 to the enhancers of *CSF1*, *EDN1*, *TGFB2*, and *VTN* to activate transcription of the corresponding genes in MCF7 (L/L) cells.

### 2.4 Increased eRNA Transcription Promotes Enhancer Activation and Cognate Gene Expression in Nutrient‐Starved Cells and Is Susceptible to ASO‐Mediated Perturbation

**FIGURE 4:**
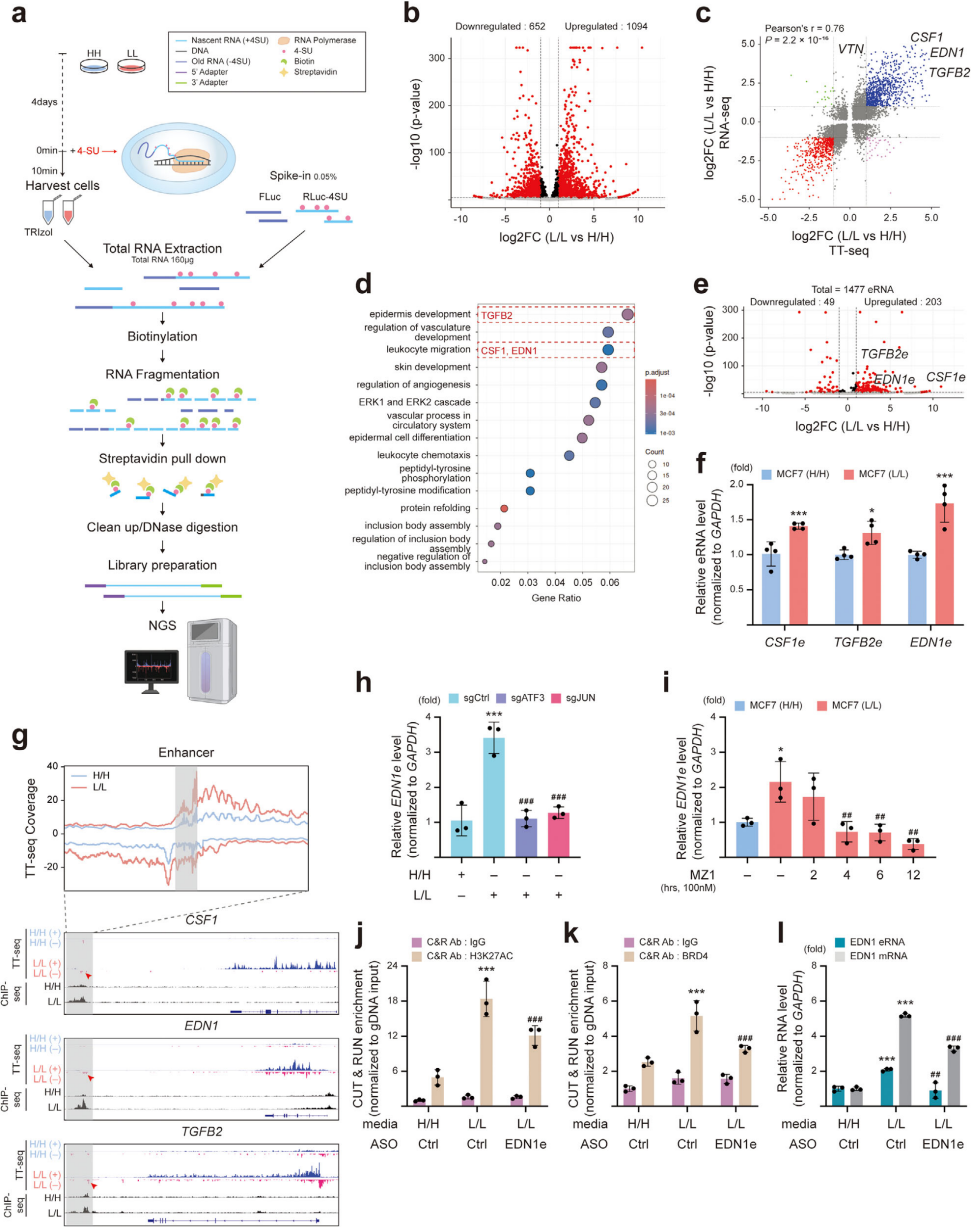
Modulation of enhancer states by eRNA transcription during nutrient starvation. a) Sample preparation for TT‑seq to identify eRNAs. b) Volcano plot showing differentially expressed genes between MCF7 (H/H) and MCF7 (L/L) cells identified from TT‑seq data, with the number of significant genes (*p* < 0.001 and |log_2_FC| > 2). c) Scatter plot of FC in differentially expressed genes between MCF7 (H/H) and MCF7 (L/L) cells from TT‑seq (X‑axis) versus RNA‑seq (Y‑axis) data, with the Pearson correlation coefficient (*r*) and corresponding significance (*p*‐value). d) Dot plot of top 15 enriched GO biological process terms for genes upregulated in MCF7 (L/L) cells (log_2_FC > 2, false discovery rate < 0.05) from TT‑seq data. Dot size reflects gene count and color shows adjusted *p*‑value (‐log_10_ scale). e) Volcano plot showing differentially expressed eRNA identified from TT‑seq data between MCF7 (H/H) and MCF7 (L/L) cells, with the number of significant genes (*p* < 0.001 and |log_2_FC| > 2). f) RT‑qPCR with primers targeting the indicated eRNAs in MCF (H/H) and MCF7 (L/L) cells. Data are presented as mean ± SD (*n* = 3). ***p < 0.001, *p < 0.05 (Student's *t*‐test; compared with MCF7 (H/H) group). g) Line plot showing coverage [reads per kilobase per million mapped reads (RPKM)] of RNA transcribed from both forward and reverse strands across a ±3 kb window around MCF7 (L/L)‐unique enhancers (highlighted in grey) (top) and Integrated Genomic Viewer snapshot showing TT‑seq and ChIP‑seq coverage (RPKM) at selected genes and their corresponding enhancers (highlighted in grey, below). h) RT‐qPCR of MCF7 cells transfected with sgCtrl, sgATF3, or sgJUN and cultured in H/H or L/L medium for 4 d, using primers targeting the indicated eRNA. Data are presented as mean ± SD (*n* = 3). ***^, ###^ *p* < 0.001 [one‐way analysis of variance (ANOVA); *** compared with sgCtrl/HH; ### compared with sgCtrl/LL]. i) RT‐qPCR of MCF7 (H/H) and MCF7 (L/L) cells treated with MZ1 (100 nM) for the indicated durations, using primers targeting the indicated eRNAs. Data are presented as mean ± SD (*n* = 3). *p < 0.05, ^##^ *p* < 0.005 [one‐way ANOVA; * compared with MCF7(H/H); ## compared with MCF7(L/L)/MZ1(‐)] j, k) CUT&RUN–qPCR showing H3K27ac (j) and BRD4 (k) enrichment at *EDN1* enhancer in MCF7 cells transfected with ASO‐ctrl or ASO‐EDN1e and cultured in H/H or L/L medium for 4 d. *IgG* serves as negative control. Data are presented as mean ± SD (*n* = 3). ***, ^###^ *p* < 0.001 (one‐way ANOVA; *** compared with H/H‐Ctrl; ### compared with L/L‐Ctrl). l) RT‐qPCR of MCF7 cells transfected with ASO‐Ctrl or ASO‐EDN1e and cultured in H/H or L/L medium for 4 d, using primers targeting *EDN1* mRNA or *EDN1e* eRNA. Data are presented as mean ± SD (*n* = 3). ***^, ###^ *p* < 0.001, ^##^ *p* < 0.005 (one‐way ANOVA; *** compared with corresponding group in H/H‐Ctrl; ##, ### compared with corresponding groups in L/L‐Ctrl).

eRNAs are key players in enhancer activation, whereby enhancer regions are independently transcribed into short or long non‐coding RNAs [19]. To examine whether enhancer activation in MCF7 (L/L) cells is accompanied by differential eRNA expression, we performed transient transcriptome sequencing (TT‐seq) on MCF7 (H/H) and MCF7 (L/L) cells, as detailed in the Supplementary Materials and Methods (Figure 4a). TT‐Seq detects nascent RNA including eRNAs using a short pulse of 4‐thiouridine (4‐SU). Before library preparation for sequencing, we validated 4‑SU incorporation into nascent RNA by dot‑blot analysis of biotinylated RNA (Figure S4a). Furthermore, qPCR showed strong enrichment (≈300‑fold) of the 4‑SU‑containing Renilla luciferase spike‑in RNA compared with the 4‑SU‑free Firefly luciferase spike‑in RNA, confirming the specific capture of nascent 4‑SU‑labeled transcripts (Figure S4b). The library enriched with nascent RNA was subsequently used for high‐throughput sequencing. Despite the small sample size (*n* = 2), the suitability of the data for downstream analysis was confirmed by consistent sample clustering in principal component analysis and appropriate dispersion estimates (Figure S4c,d). To validate the ability of TT‐seq to capture transcriptional changes, we performed differential expression analysis, identifying 1,094 upregulated and 652 downregulated genes (Figure 4b). TT‐seq identified a substantially higher number of differentially expressed genes than steady‐state RNA‐seq (Figure 1h), reflecting its superior sensitivity to rapid changes in nascent transcriptional activity. TT‐seq‐based gene expression profiles showed a significant positive correlation with those from RNA‐seq, with *CSF1*, *EDN1*, and *TGFB2* consistently upregulated in both analyses (Figure 4c). Because TT‐seq directly measures nascent RNA synthesis, the elevated levels of *CSF1*, *EDN1*, and *TGFB2* in MCF7 (L/L) cells resulted from increased transcriptional output, suggesting that these genes are subject to enhancer‐mediated transcriptional control. Compared with RNA‐seq, GO analysis showed partially distinct enrichment patterns in TT‐Seq (Figures 1j and 4d). Nevertheless, *CSF1*, *EDN1*, and *TGFB2* were well represented within these ontology terms (Figure 4d). To characterize differential eRNA expression, we merged Binary Alignment/Map files from MCF7 (H/H) and MCF7 (L/L) replicates to define consensus eRNA coordinates and identified 1,477 consensus eRNA regions across both cell lines using an established procedure (Figure S4e). Differential expression analysis revealed that 203 eRNAs were upregulated and 49 were downregulated in MCF7 (L/L) cells relative to MCF7 (H/H) cells (Figure 4e). Notably, eRNA transcription originating from the *CSF1*, *TGFB2*, and *EDN1* enhancers was increased in MCF7 (L/L) cells (Figure 4e). RT‐qPCR confirmed increased eRNA levels of *CSF1e*, *TGFB2e*, and *EDN1e* in MCF7 (L/L) cells (Figure 4f). Furthermore, *EDN1e* eRNA expression was significantly increased in MDA‐MB‐231 (L/L) cells compared with that in MDA‐MB‐231 (H/H) cells (Figure S4f). To determine whether enhanced eRNA transcription is a gene‐specific event or broader regulatory feature, we analyzed TT‐seq signals across MCF7 (L/L)‐unique enhancers. We observed a global increase in bidirectional eRNA synthesis, spanning both sense and antisense strands, in MCF7 (L/L) cells compared with that in MCF7 (H/H) cells, suggesting that nutrient deprivation facilitates a pervasive upregulation of eRNA transcription across MCF7 (L/L)‐unique enhancers (Figure 4g, upper). Integrative analysis of H3K27ac ChIP‐seq with TT‐seq data confirmed the consistent increase in eRNA synthesis from the *CSF1*, *TGFB2*, and *EDN1* enhancer regions in MCF7 (L/L) cells relative to MCF7 (H/H) cells. This suggests that starvation‐induced activation of *CSF1*, *EDN1*, and *TGFB2* enhancers is accompanied by increased eRNA transcription from corresponding enhancers (Figure 4g, lower). Given that it showed the most pronounced induction in MCF7 (L/L) cells, we selected *EDN1e* for subsequent functional analysis. To test whether *EDN1e* transcription is regulated by enhancer‐binding TFs, we measured *EDN1e* levels in ATF3‐ and JUN‐knockout MCF7 cells. RT‐qPCR revealed that depletion of either ATF3 or JUN significantly attenuated the induction of *EDN1e* in MCF7 (L/L) cells, confirming that both factors are needed for *EDN1e* transcription (Figure 4h). Consistently, MZ1 treatment reduced *EDN1e* eRNA expression in MCF7 (L/L) cells in a time‐dependent manner (Figure 4i). Together, these data showed that enhancer activation is required for eRNA transcription. Vice versa, to assess the importance of eRNA for enhancer activation, we designed ASOs targeting *EDN1* eRNA (Table S3). We used four commercially synthesized gapmer ASOs targeting bidirectional EDN1 eRNAs transcribed from both the coding and non‐coding strands (Figure S4g). Transfection of ASO effectively reduced its target eRNA expression (Figure S4h). We pooled all four ASOs to maximize knockdown efficiency. The pooled mixture, hereafter referred to as ASO, significantly reduced *EDN1* mRNA levels in wild‐type MCF7 cells cultured under L/L conditions but had no effect in EDN1 enhancer‐deleted MCF7 cells, indicating that ASO‐mediated suppression of *EDN1* mRNA depends on eRNA expression (Figure S4i). Transfection of these ASOs into MCF7 cells reduced p300 occupancy, followed by decreased H3K27ac levels and BRD4 recruitment at the EDN1 enhancer (Figure S4j and Figure 4j,k), resulting in reduced *EDN1* mRNA expression (Figure 4l), suggesting that silencing of eRNA expression reduces the enhancer function. Consistently, transfection of ASO significantly reduced *EDN1* mRNA expression in MDA‐MB‐231 (L/L) cells (Figure S4k). Together, these data demonstrate that eRNAs play an important role in enhancer‐driven gene expression, as exemplified by *EDN1e* eRNA.

### 2.5 Inhibition of BRD4‐Dependent EDN1 Overexpression Suppresses Invasive Phenotypes and Metastatic Dissemination in Breast Cancer Cells

**FIGURE 5:**
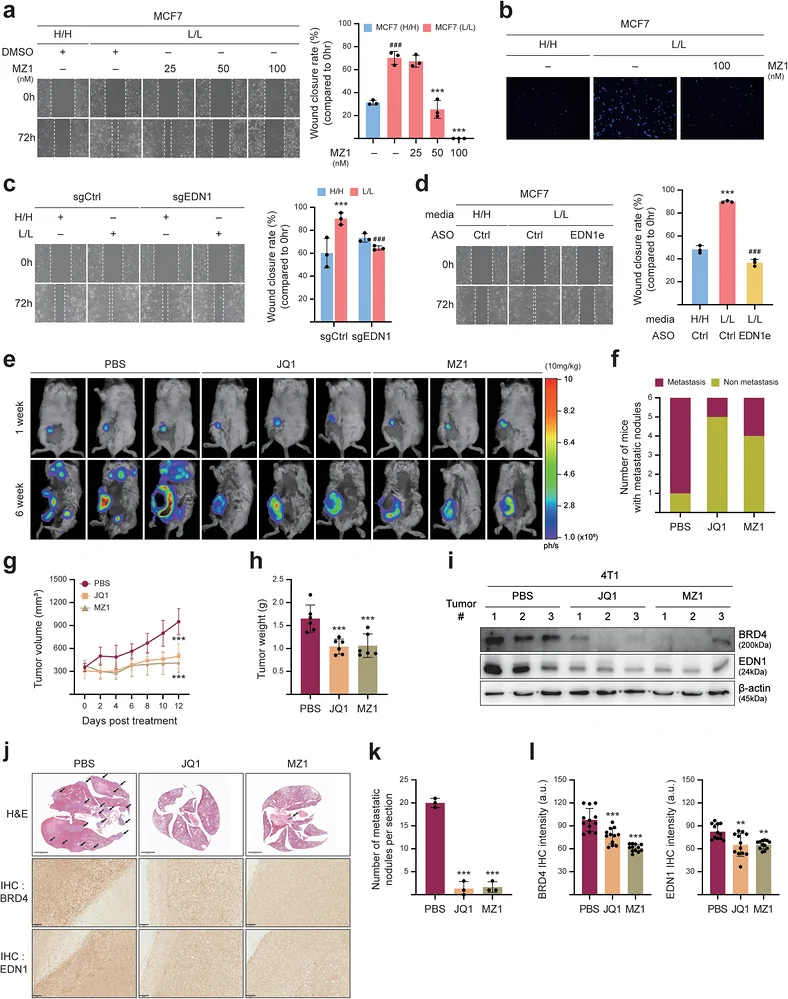
BRD4‑dependent regulation of EDN1 in breast cancer cell invasiveness and metastasis. a) Representative images of wound‑healing assay with MCF7 (H/H) and MCF7 (L/L) cells treated with the indicated concentration of MZ1 for 72 h (left). Wound closure rate is expressed as the percentage of closure at 72 h relative to 0 h (right). Data are presented as mean ± SD (*n* = 3). ^***^, ^###^ *p* < 0.001 [one‐way ANOVA; ### compared with MCF7 (H/H), *** compared with MCF7 (L/L)/MZ1(‐)]. b) Representative images of Transwell invasion assay with MCF7 (H/H) and MCF7 (L/L) cells treated with 100 nm MZ1 for 72 h, with nuclear 4′,6‐diamidino‐2‐phenylindole staining (blue). c) Representative images of wound‐healing assay with MCF7 wild‐type (sgCtrl) and *EDN1*‐knockout (sgEDN1) cells cultured in either H/H or L/L medium for 4 d (left). Wound closure rate is expressed as in panel (a) (right). Data are presented as mean ± SD (*n* = 3). ^***^, ^###^ *p* < 0.001 (one‐way ANOVA; compared with corresponding H/H samples in each group). d) Representative images of wound‑healing assay with MCF7 cells transfected with ASO‐Ctrl or ASO‐EDN1e and cultured in either H/H or L/L medium for 4 d (left), with quantified wound closure rate (%). Data are presented as mean ± SD (*n* = 3). ^***^, ^###^ *p* < 0.001 (one‐way ANOVA; *** compared with H/H; ### compared with L/L‐Ctrl). e) Representative in vivo bioluminescence images of BALB/c mice bearing orthotopic 4T1‐luc tumors treated with JQ1 or MZ1. Ventral views at 1‐week post‐implantation (pre‐treatment baseline) and after 6 weeks of treatment in the same cohort of mice. Color scale indicates photon flux (photons/s). f) Stacked bar plot showing metastatic incidence in mice after 6 weeks of treatment. g) Tumor growth curves showing changes in tumor volume after treatment with JQ1 or MZ1 for the indicated number of days. Data are presented as mean ± SD (*n* = 6). ***p < 0.001(Two‐way ANOVA, compared with phosphate‐buffered saline (PBS) group). h) Weights of 4T1‐luc‐derived tumors after 6 weeks of treatment with JQ1 or MZ1. Data are presented as mean ± SD (*n* = 6). ***p < 0.001 (one‐way ANOVA, compared with PBS group). i) Western blot analysis of JQ1‐ or MZ1‐treated tumor lysates using the indicated antibodies. j) Histochemical examination of tumor specimens. Representative hematoxylin‐and‐eosin‐stained images showing metastatic nodules (arrows) in lung sections from BALB/c mice harboring orthotopic 4T1‐luc tumors after 6 weeks of JQ1 or MZ1 treatment (upper), and nodules quantified as proportion of nodules per field (right). Representative IHC images of BRD4 and EDN1 expression in primary 4T1‐luc tumors after JQ1 or MZ1 treatment (lower). k, l) Quantification of metastatic nodules in lung tissue (k) and BRD4 or EDN1 IHC intensity in primary tumors (l). Data are presented as mean ± SD (*n* = 3–12). ***p < 0.001, **p < 0.005 (one‐way ANOVA, compared with PBS group).

We assessed the contribution of enhancer‐driven *EDN1* upregulation to breast carcinogenesis through functional assays focused on cell migration and metastasis. In wound healing assays, the wound closure rate was significantly higher in MCF7 (L/L) than in MCF7 (H/H) cells, and was reduced by MZ1 treatment (Figure 5a). Consistently, wound closure rate in MDA‐MB‐231 (L/L) cells was increased compared with that in MDA‐MB‐231 (H/H) cells and significantly reduced by MZ1 treatment (Figure S5a). Furthermore, MZ1 treatment reduced the enhanced Transwell migration of MCF7 (L/L) cells (Figure 5b). We examined whether increased EDN1 expression induced by BRD4‐dependent enhancer activation promotes cell migration and invasion through knockout of *EDN1* in MCF7 cells using CRISPR/Cas9 (Figure S5b). *EDN1* knockout reduced the elevated wound closure rate of MCF7 cells in L/L medium, suggesting that EDN1 plays an essential role in promoting the invasive phenotype of MCF7 cells under nutrient‐deprived conditions (Figure 5c). To determine the contribution of other candidate genes to the migration phenotype, we independently knocked out *CSF1*, *TGFB2*, and *VTN* (Figure S5c). Knockout of each gene reduced the wound closure rate to varying degrees, suggesting that CSF1, TGFB2, and VTN also contribute to cell migration, with EDN1 as a representative mediator among these candidates (Figure S5d). Transfection of ASOs targeting *EDN1* eRNA reduced the enhanced migration of MCF7 (L/L) and MDA‐MB‐231 (L/L) cells (Figure 5d and Figure S5e), confirming the role of *EDN1e* eRNA in cell migration. Furthermore, increased wound healing in MCF7 cells induced by nutrient deprivation was attenuated by deletion of the *EDN1* enhancer, suggesting the role of enhancer‐dependent *EDN1* expression in cell migration (Figure S5f). In 3D spheroid culture, MZ1 treatment reduced spheroid formation by MCF7 (L/L) cells relative to MCF7 (H/H) cells (Figure S5g). Together, these findings demonstrate that enhancer activation promotes cell migration through the upregulation of specific target genes, with the *EDN1* enhancer serving as a representative example. To elucidate the role of BRD4‐dependent enhancer activation and *EDN1* expression in metastasis in vivo, luciferase‐labeled 4T1 cancer cells were inoculated into mouse mammary ducts and treated with a BRD4 inhibitor (JQ1) and MZ1. Tumor progression and metastatic burden were monitored using bioluminescence imaging. Although tumors continued to grow in all treatment groups, metastasis was strongly inhibited in the JQ1‐ (5/6 vs 1/6 mice) and MZ1‐treated (4/6 vs 1/6 mice) models (Figure 5e,f). We observed a decrease in tumor volume during the course of JQ1 and MZ1 treatment (Figure 5g). At the experimental endpoint, tumors were excised and weighed. Treatment with JQ1 and MZ1 significantly reduced tumor weight compared with that in the control treatment (Figure 5h and Figure S5h). To demonstrate the association between metastasis and increased BRD4 and EDN1 expression, we examined their protein levels in primary tumor samples using western blot analysis. EDN1 and BRD4 expression decreased following treatment with JQ1 and MZ1 (Figure 5i). Histochemical analysis of lung sections with hematoxylin and eosin staining showed that JQ1 and MZ1 substantially reduced the number of metastatic nodules in lung tissue (Figure 5j, upper; Figure 5k). Immunohistochemistry (IHC) confirmed that BRD4 and EDN1 expression was reduced in primary tumors following JQ1 and MZ1 treatment (Figure 5j, lower; Figure 5l). Together, these in vivo findings demonstrate that pharmacological BRD4 inhibition reduces EDN1 expression and strongly restricts the pulmonary metastatic colonization of breast cancer cells, highlighting its therapeutic potential in targeting nutrient‐deprived tumor microenvironments.

### 2.6 BRD4 Expression Positively Correlates with EDN1 in Metastatic Breast Cancer, and Its Targeting Suppresses Tumorigenesis in Xenograft Model

**FIGURE 6:**
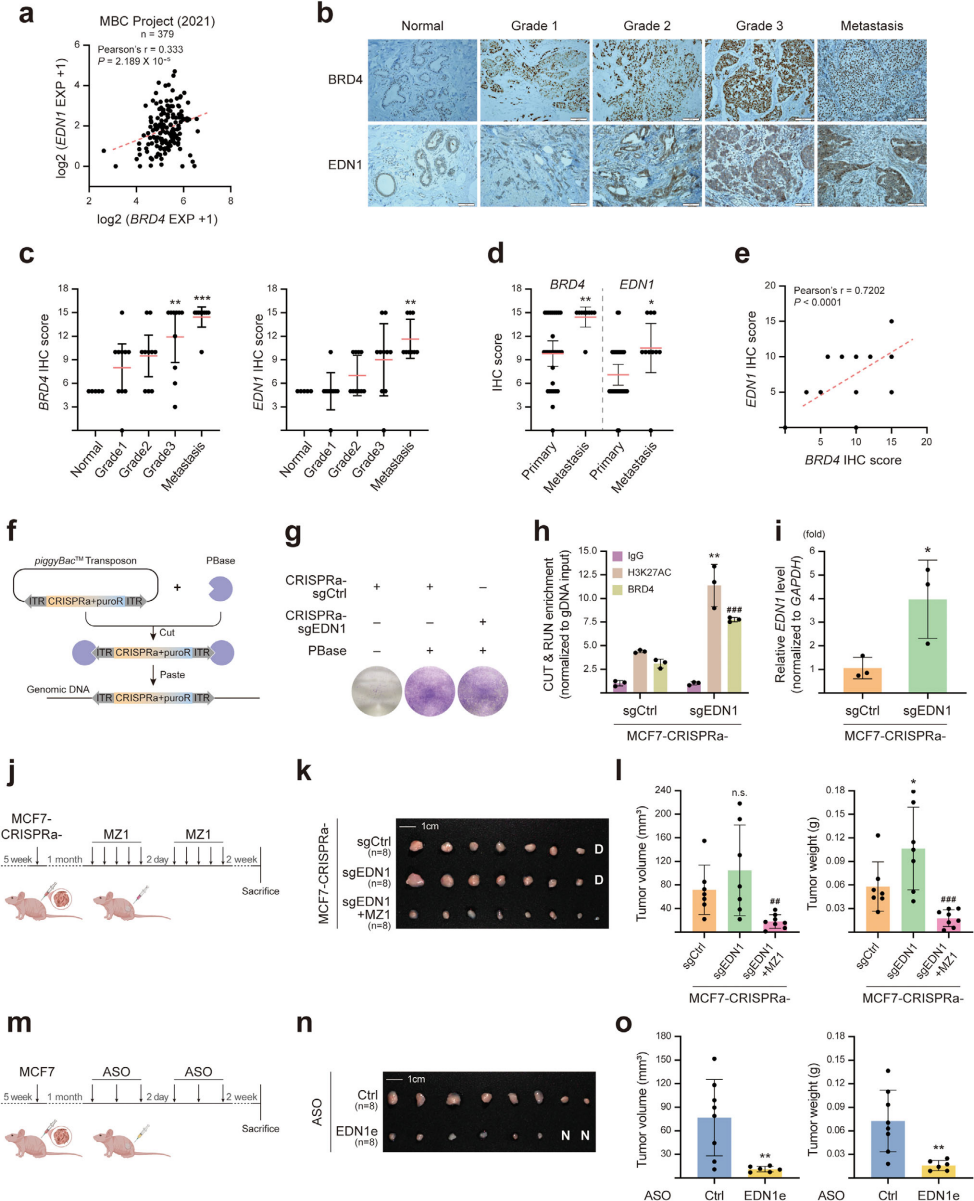
BRD4 and EDN1 expression in metastatic breast cancer and xenograft tumor development. a) Correlation between *BRD4* and *EDN1* mRNA expression from Metastatic Breast Cancer Project (2021, available from cbioportal.org) dataset. Pearson's correlation (*r*) and significance (*P‐*value) are indicated. b) Representative IHC staining images of BRD4 and EDN1 in human breast cancer primary tumors (grade I–III) and lymph node metastases. c) IHC scoring of BRD4 (left) and EDN1(right) based on tumor progression and metastasis. Data are presented as mean ± SD (*n* = 10). **p < 0.005, ***p < 0.001 (one‐way ANOVA; compared with Normal). d) IHC scoring of BRD4 and EDN1 in primary tumors and metastases. Data are represented as mean ± SD (*n* = 30 primary, *n* = 10 metastases). **p < 0.005, *p < 0.05 (one‐way ANOVA; compared with Primary). e) Correlation between BRD4 and EDN1 IHC scores in metastasized breast tumor specimen. Pearson's correlation (*r*) and significance (*p*‐value) are indicated. f) Establishment of *EDN1* enhancer‐targeting CRISPRa cell line with PiggyBac system. g) Colony formation of stable puromycin‐resistant MCF7‐CRISPRa cells cultured in the presence of 2 µg mL^−1^ puromycin for 1 week. h) CUT&RUN–qPCR analysis of MCF7‐CRISPRa cells expressing sgCtrl or sgEDN1 using the indicated antibodies followed by qPCR with primers targeting the *EDN1* enhancer region. Data are presented as mean ± SD (*n* = 3). **p < 0.005, ^###^ *p* < 0.001 (one‐way ANOVA; compared with corresponding samples in sgCtrl) i) RT‐qPCR of *EDN1* mRNA expression in MCF7‐CRISPRa cells expressing sgCtrl or sgEDN1. Data are presented as mean ± SD (*n* = 3). *p < 0.05 (Student's *t*‐test). j‐l) In vivo experimental timeline, including mouse acclimatization, MCF7 xenograft implantation, and MZ1 treatment, as detailed in Supplementary Materials and Methods (j); images of tumors (k); and tumor weight and volume measured at the experimental endpoint (l). “D” indicates mice that died during the experiment. Data are presented as mean ± SD (*n* = 8). n.s., non‐significant, *p < 0.05, ^##^ *p* < 0.005, ^###^ *p* < 0.001 (one‐way ANOVA; * compared with sgCtrl; ^##, ###^ compared with sgEDN1). m–o) In vivo experimental timeline, including mouse acclimatization, MCF7 xenograft implantation, and ASO treatment, as detailed in Supplementary Materials and Methods (m); images of tumors (n); and tumor weight and volume measured at the experimental endpoint (o). “N” indicates that no tumor could be recovered due to its small size. Data are presented as mean ± SD (*n* = 8). **p < 0.005 (one‐way ANOVA; compared with Ctrl).

To explore the clinical relevance of BRD4‐dependent EDN1 expression in breast cancer metastasis, we analyzed mRNA expression profiles from the Metastatic Breast Cancer Project dataset (Figure 6a). A significant positive correlation was observed between *BRD4* and *EDN1* expression, providing clinical evidence for a coordinated regulatory relationship in metastatic cases. Consistently, a positive correlation was observed between *BRD4* and *CSF1*, *TGFB2*, and *VTN* expression (Figure S6a). In patients with breast cancer, IHC showed that both BRD4 and EDN1 expression increased with tumor grade and was highest in the metastatic lymph nodes (Figure 6b,c). Both BRD4 and EDN1 were commonly upregulated in metastatic specimens compared with that in primary tumor samples (Figure 6d). Furthermore, a significant positive correlation was observed between EDN1 and BRD4 IHC scores (Figure 6e). A similar pattern was observed for CSF1, suggesting similar expression profiles for other candidate genes in advanced metastatic cases (Figure S6b,c). Together, these data show a strong positive relationship between BRD4 and EDN1 expression during metastatic progression in breast cancer and also suggest that other candidate genes follow similar expression patterns in this breast cancer cohort.

To assess the in vivo relevance of enhancer‐dependent EDN1 regulation, we examined the *EDN1e* eRNA expression in the core and peripheral regions of MCF7 xenograft tumors. The results showed that *EDN1e* level was also significantly elevated in the tumor core (Figure S6d). Consistent with the locally reduced glucose and glutamine levels (Figure 1a), increased ATF3, c‐JUN, and EDN1 expression was observed in the tumor core compared with that in the tumor periphery (Figure S6e) supporting the physiological relevance of the enhancer‐dependent mechanism.

We directly determined the role of enhancer‐driven EDN1 overexpression on breast tumorigenesis in MCF7‐CRISPRa cells. We achieved stable integration of the dCas9–p300 fusion gene and *EDN1* enhancer‐targeting guide RNAs using PiggyBac‐mediated transposition (Figure 6f,g). CUT&RUN–qPCR confirmed that H3K27ac modification and BRD4 binding at the *EDN1* enhancer were significantly increased in CRISPRa cells expressing sgEDN1‐enh compared with those expressing sgCtrl (Figure 6h). This was accompanied by elevated *EDN1* mRNA levels (Figure 6i). These MCF7‐CRISPRa cells were implanted into immunosuppressed nude mice, followed by intraperitoneal MZ1 treatment (Figure 6j). The weights of tumors formed by MCF7‑CRISPRa cells expressing sgEDN1‐enh were significantly higher than those formed by sgCtrl‐expressing cells. Tumor weight and volume were reduced upon treatment with MZ1 (Figure 6k,l). This indicates that enhanced tumor formation in sgEDN1‐enh‐expressing cells depends on BRD4‑mediated enhancer activation. As an alternative approach to inhibit EDN1 expression induced by enhancer activation, we administered an ASO cocktail targeting *EDN1* eRNA into nude mice harboring MCF7 cell‐derived tumors (Figure 6m), which significantly reduced both tumor volume and weight compared with those in the control ASO treatment (Figure 6n,o). Western blotting showed elevated EDN1 expression in tumors derived from sgEDN1‐enh‑expressing CRISPRa cells, which was diminished by MZ1 treatment (Figure S6f). Meanwhile, EDN1 expression was reduced in tumors treated with EDN1‑targeting ASO (Figure S6g). Together, these data directly demonstrate the role of enhancer‐driven EDN1 overexpression in breast tumorigenesis.

## 3 Discussion

**FIGURE 7:**
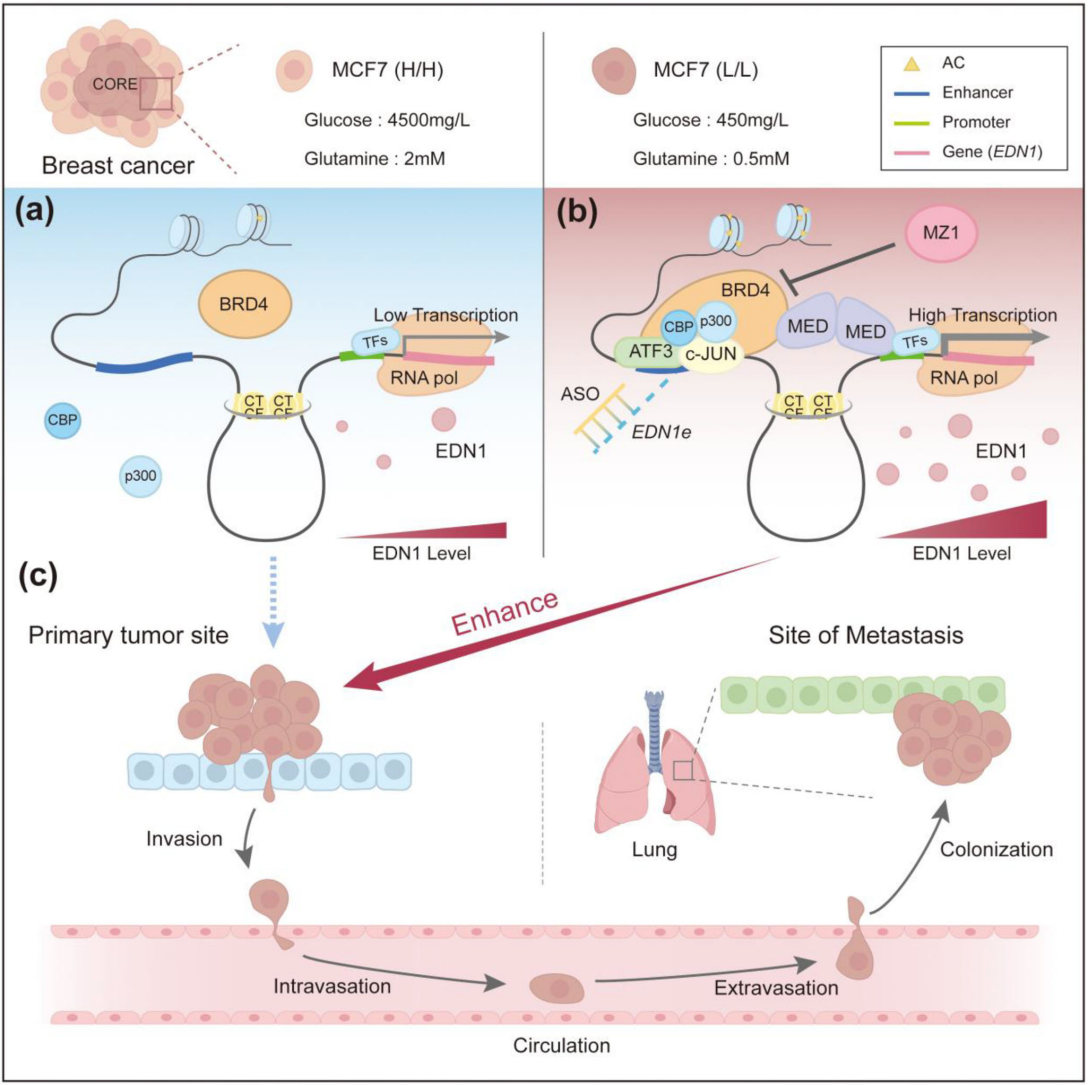
Proposed model of BRD4‐dependent enhancer reprogramming driving breast cancer progression and lung metastasis. a, b) To model nutrient‐replete and nutrient‐restricted tumor core conditions, we established MCF7 (H/H) (a) and MCF7 (L/L) (b) cells through adaptation to high‐glucose–high‐glutamine and low‐glucose–low‐glutamine culture media, respectively. In MCF7 (L/L) cells, overexpression of the TFs ATF3 and c‐JUN enhances the recruitment of BRD4 to specific enhancer sequences. Together with mediator complex (MED) proteins, this promotes assembly of an active enhancer complex that facilitates transcription of enhancer regions into eRNAs, which further stabilize and reinforce enhancer activity. Consequently, enhanced enhancer assembly drives the expression of target genes such as *EDN1*. Disruption of enhancer complex assembly using MZ1, a PROTAC degrader of BRD4, or targeting eRNAs with ASOs destabilizes the enhancer and reduces *EDN1* expression. c) At the phenotypic level, increased EDN1 expression promotes the invasiveness of MCF7 (L/L) cells. Treatment with MZ1 or ASOs reduces EDN1 expression in 4T1 breast cancer cells and suppresses lung metastasis.

Nutrient scarcity is a critical selective pressure that drives cellular heterogeneity and tumor evolution. Understanding the adaptation of cancer cells to nutrient deprivation is important for designing effective targeted therapies. Here, we showed that glucose–glutamine restriction induces extensive enhancer remodeling and eRNA activation in breast cancer cells, identifying an epigenetic link between metabolic stress and prometastatic gene expression. We identified BRD4 as the central mediator of this enhancer‐dependent transcriptional response and demonstrated that PROTAC‐induced BRD4 degradation or inhibition with JQ1 effectively suppresses metastatic progression in breast cancer models. We revealed stress‐induced eRNAs as functional regulators of enhancer activity and showed that ASO‐mediated targeting of *EDN1* eRNA reduces target gene expression and in vivo breast tumorigenesis. In clinical samples, metastatic cases showed increased BRD4 and EDN1 expression, consistent with the in vitro experimental findings. Our findings define metabolic stress‐responsive enhancers and their regulators as promising therapeutic targets for blocking nutrient‐driven metastatic evolution in breast cancer (Figure 7).

Compared with normal cells, cancer cells have distinct metabolic characteristics. Their rapid and uncontrolled proliferation creates high metabolic demand that often depletes local nutrients, particularly glucose and glutamine [5]. To survive under nutrient‐limited conditions, cancer cells adopt adaptive strategies such as invasion and metastasis to nutrient‐rich niches [31]. The phenotypic transitions require widespread changes in gene expression. However, the transcriptional and epigenetic responses to glucose and glutamine starvation remain poorly understood in breast cancer.

In this study, we established a breast cancer cell model adapted to transient nutrient deprivation. We chose transient rather than prolonged deprivation, as described by Tsai et al. [7], to capture reversible and epigenetically driven adaptive mechanisms while minimizing confounding effects from genetic mutations or chromosomal aberrations that may arise during long‐term culture. A key finding was that cells exposed to transient nutrient deprivation exhibit increased proliferation compared with those continuously cultured under high‐nutrient conditions when transferred to fresh media. Although cells grown under low‐nutrient conditions generally do not outperform those grown under nutrient‐rich conditions, adaptation or pre‐conditioning to transient deprivation enhances survival, migration, and rebound proliferation upon nutrient replenishment [7, 32]. These findings have important clinical implications since they help explain why cancer cells become more aggressive and proliferative following episodes of nutrient deprivation [7, 33].

From a therapeutic perspective, it is crucial to identify the activated transcriptional programs under nutrient starvation. ChIP‐seq showed that global gene transcription was reduced, as reflected by a decrease in the H3K27ac signal (a hallmark of active transcription) across gene promoters. This is expected since low nutrient availability makes broad non‐essential transcription energetically unfavorable. Despite this global reduction in active promoter levels, the expression of specific genes involved in ECM remodeling, migration, and invasion increased following nutrient starvation. Similar findings were observed for fibroblasts and breast cancer cells under serum starvation [34, 35], whereas evidence linking glucose and glutamine deprivation to invasion is lacking. For many of the upregulated genes, we failed to detect substantially increased H3K27ac levels in their promoter regions. This raises the question of how transcription was enhanced despite reduced promoter acetylation. Acetylation is not strictly required for promoter activation because enhancers can drive transcription through redundant chromatin loop formation [36]. To identify these enhancers, we analyzed the distribution of H3K27ac peaks unique to starved cells and found that most were located in non‐promoter regions, consistent with enhancer activation.

Enhancer activation requires the cooperative action of histone acetyltransferases, including p300/CBP, and epigenome readers, such as BRD4 [36, 37]. CBP and p300 protein levels decreased in the nutrient‐starved cells. Although this explains the global reduction in acetylation across promoters, it also raises an important question: How does acetylation increase at certain enhancer sequences under the same conditions? CBP/p300 may be recruited to these sites via interactions with TFs [38]. Upon binding to TFs, the histone acetyltransferase domain is activated [39]. This process may be further enhanced by stress‐associated signaling pathways [40], leading to increased H3K27ac modification at enhancers. H3K27ac in the promoters of p53 target genes remained unchanged or even increased following p300 knockdown in mouse embryonic stem cells [41]. This highlights a paradoxical mechanism that favors the prioritization over the protein level of CBP/p300. Binding of eRNA to CBP stimulates its catalytic activity and increases H3K27ac modification [42]. Although our study did not delineate the precise mechanism, we showed that H3K27ac levels in specific enhancers were increased despite reduced CBP/p300 expression. Furthermore, p300–enhancer binding was increased in nutrient‐starved cells. Since several TFs, including AP‐1 family members, are specifically overexpressed in nutrient‐deprived cells, they may recruit p300/CBP to specific enhancers to maintain H3K27ac levels despite the global reduction of CBP/p300 expression. CBP/p300‑induced histone acetylation forms a docking site for BRD4 binding. In our study, BRD4 expression increased in starved cells, which may have been recruited to enhancers via direct binding with acetylated histones. Specific TFs may also recruit BRD4 to the enhancer given that the TF‐binding sites occur within the enhancer sequence [43]. Increased binding of H3K27ac and BRD4 to enhancers reinforces their ability to activate target gene expression.

Although H3K27ac modification and BRD4 recruitment are essential for enhancer activation, their functional importance lies in their ability to induce target gene expression. Conventionally, enhancer sequences have been cloned into plasmid constructs to assess their ability to drive reporter gene expression upon transfection into host cells [44]. While this approach demonstrates whether a given sequence can function as an enhancer, it does not capture the complexity of endogenous enhancer–promoter interactions. With advances in CRISPR/Cas9‐based epigenome editing, it is possible to induce the histone acetylation of native enhancers [45]. Using the dCas9–p300 fusion, we targeted selected enhancers and confirmed the activation of their corresponding genes. This approach bypasses TF binding but still requires BRD4 recruitment, as verified by CUT&RUN analysis. A similar mechanism is activated in nutrient‐deprived breast cancer cells, in which TF binding induces the expression of metastasis‐associated genes via CBP/p300–BRD4 recruitment.

Given the central importance of TFs in the formation and maintenance of enhancers and enhancer‐driven transcriptional programs in starved cells, we focused on their binding landscape in enhancer regions. Binding sites for AP‐1 TF family members were preferentially enriched within enhancer sequences unique to starved cells. Several AP‑1 components, including JUN and ATF3, were highly overexpressed according to RNA‑seq. ATF3 and JUN are well‑established stress‑responsive TFs that promote the expression of survival‑ and adaptation‑related genes [35, 46]. However, no studies have examined the binding of these factors to enhancer sequences in the context of nutrient deprivation. Our results suggest that AP‑1 factors, particularly ATF3 and JUN, bind to and activate these enhancers, as demonstrated by CUT&RUN analysis, establishing their role in starvation‐induced enhancer formation.

eRNAs are another key component of active enhancers [19]. eRNAs help stabilize and activate enhancers by reinforcing enhancer–promoter looping and recruiting coactivators. They can support cohesin occupancy at enhancers, which strengthens chromatin looping. m^6^A‐modified eRNAs can recruit YTHDC1 to promote BRD4 condensate formation, helping build phase‐separated transcriptional hubs that sustain enhancer activity and target gene expression [19, 47]. Because eRNAs are rapidly degraded and poorly captured by conventional RNA‐seq, we performed TT‑seq to capture nascent transcription events and identified several eRNAs, including *EDN1* eRNA. Depletion of *EDN1* eRNA using ASOs reduced H3K27ac and BRD4 occupancy at the enhancer site, suppressing EDN1 expression in cell cultures and tumorigenesis in mouse models. Although we demonstrated that *EDN1* eRNA is essential for enhancer activation and *EDN1* gene expression, the precise mechanism requires further investigation. In addition to ASOs, we showed that the inhibition of BRD4 using chemical inhibitors, such as JQ1, or degraders, such as the PROTAC MZ1, can prevent the metastatic dissemination of breast cancer cells in mice. Although BRD4 inhibitors are appealing as first‐line therapeutic agents, they are associated with high levels of systemic toxicity and rapid emergence of drug resistance. Therefore, ASOs may be an attractive alternative or complementary strategy for targeting enhancer‐driven transcription in cancer cells.

Regarding the limitations of this study, we did not assess the metastatic behavior of MCF7 cells in the xenograft model because nude mice are not suitable for metastasis studies [48]. The estrogen dependence of MCF7 cells complicates the establishment of large tumors in vivo. Instead, we used 4T1 cells, which are known to form large primary tumors with high metastatic potential. Although controlling glucose and glutamine levels within the tumor microenvironment is difficult, nutrient‐deprived regions are likely to develop spontaneously, similar to human tumors. The cells in these regions may acquire higher metastatic capacities [49]. Therefore, we allowed the tumors to reach a large size, that is, ≈300 mm^3^, before initiating the BRD4 inhibitor treatment. Consistent with our expectations, the inhibitor suppressed metastasis despite the relatively large tumor size, underscoring the preclinical relevance of our findings. Future studies should evaluate the comprehensive safety profile (e.g., blood counts, liver/kidney functions) and therapeutic efficacy of ASOs against metastasis using more relevant clinical models. Furthermore, our ASO‐based targeting approach was limited to *EDN1e*. The development and validation of ASOs targeting additional candidate eRNAs will provide a more comprehensive understanding of the role of eRNAs in enhancer regulation during nutrient deprivation. To assess the clinical relevance of our findings, we analyzed breast cancer samples and found that both BRD4 and EDN1 expression was frequently upregulated in tumors with lymph node metastasis. Although these data are consistent with our experimental results, suggesting that BRD4 regulates EDN1 expression, they only represent a correlation. Owing to limited sample size, we could not establish the prognostic significance of BRD4 and EDN1 in breast cancer metastasis. Future analyses should consider larger cohorts stratified by BRD4 expression.

In conclusion, glucose and glutamine deprivation‐induced enhancers are dynamic drivers of breast cancer metastasis and represent clinically actionable vulnerabilities. Targeting this stress‐responsive epigenetic circuitry with BRD4 degraders and eRNA‐specific ASOs offers a precise therapeutic strategy for blocking starvation‐driven metastatic evolution.

## Author Contributions

**Poshan Yugal Bhattarai**: conceptualization, investigation, validation, software, writing – review and editing, and methodology. **Arathy Vasukutty**: investigation, validation, and writing – original draft. **Yujin Na**: visualization, investigation, and formal analysis. **Sung‐Chul Lim**: investigation and resources. **Hong Seok Choi**: conceptualization, supervision, resources, project administration, funding acquisition, writing – review and editing, and data curation.

## Funding

This work was supported by the National Research Foundation of Korea (NRF), funded by the Korean government (NRF‐2022R1A2C2005094) and Ministry of Science, Information and Communications Technology (ICT), and Future Planning (NRF‐2022R1A5A2030454).

## Ethics Statement

The study was conducted according to the ARRIVE guidelines and approved by the Animal Experiments Committee of Chosun University (CIACUC 2025‐S0037). Informed consent was obtained from all participants, and the study was conducted following protocols approved by the Ethics Committee of Chosun University Hospital in accordance with the Declaration of Helsinki.

## Consent

Informed consent was obtained from all participants.

## Conflicts of Interest

The authors declare no conflicts of interest.

## Supporting information




**Supporting File**: advs77493‐sup‐0001‐SuppMat.docx.

## Data Availability

The next‐generation sequencing data generated in this study have been deposited in the NCBI Gene Expression Omnibus under accession numbers GSE330661 (RNA‐seq), GSE330662 (ChIP‐seq), and GSE330663 (TT‐seq). The codes/scripts used for the analyses are based on standard algorithms and will be made available upon reasonable request.
