# Supplementary material for: Glucose and Glutamine Deprivation Promotes Breast Cancer Lung Metastasis via BRD4‐Dependent Enhancer Activation
Source: Adv Sci (Weinh). 2026 Aug 29:e77493. Online ahead of print. doi: 10.1002/advs.77493 (PMC13525700; doi:10.1002/advs.77493)
Supplement: Supplementary file 1 [file ADVS-9999-e77493-s001.docx]

Supporting Information

**Glucose and glutamine deprivation promote breast cancer lung metastasis via BRD4-dependent enhancer activation**

*Poshan Yugal Bhattarai^#^, Arathy Vasukutty^#^, Yujin Na, Sung-Chul Lim, and Hong Seok Choi**

P.Y. Bhattarai, A. Vasukutty, Y. Na, H.S. Choi

Honam Regional Future Drug Development Convergence Education & Research Division (BK21 FOUR), College of Pharmacy, Chosun University, 61452, Jeonnam-Gwangju Special Metropolitan City, Republic of Korea

S.-C. Lim

Department of Pathology, School of Medicine, Chosun University, 61452, Jeonnam-Gwangju Special Metropolitan City, Republic of Korea

^#^These authors contributed equally to this work.

*Correspondence:

Hong Seok Choi

College of Pharmacy, Chosun University

309 Pilmun-daero, Dong-gu, Gwangju 501-759, Republic of Korea

Tel: +82 622306379

Fax: +82 622225414

E-mail: [chs@chosun.ac.kr](mailto:chs@chosun.ac.kr)

**Supplementary Materials and Methods**

**Cell culture and stable cell line establishment**

MCF7, MDA-MB-231, and HEK293 cell lines, obtained from the American Type Culture Collection (ATCC, Manassas, VA, USA), were cultured in Dulbecco’s modified Eagle’s medium supplemented with 10% fetal bovine serum (FBS) and maintained at 37 °C in a humidified atmosphere with 5% CO_2_. Under similar conditions, 4T1-luc cells (ATCC) were cultured in RPMI 1640 medium supplemented with 10% FBS. To generate MCF7-CRISPRa cell lines, MCF7 cells were transfected with 3 µg of transposon vectors encoding dCas9–p300 with either sgEDN1 or sgCtrl, along with 1 µg of hyperactive piggyBac transposase (VectorBuilder, cat. no. VB010000-9365tax, Chicago, IL, USA). For the MCF7-CRISPRa-sgEDN1 cell, a pooled mixture of three plasmids, each encoding a distinct sgRNA targeting *EDN1* enhancer, was co-transfected with the piggyBac transposase. Guide RNA sequences were designed using the CRISPick tool (Broad Institute) to minimize potential off-target effects. Forty-eight hours post-transfection, cells were subjected to selection with 2 µg mL^–1^ puromycin for 7 days. Surviving cells were pooled and expanded for subsequent experiments. To establish MCF7 (H/H) and MCF7 (L/L) cells, MCF7 cells were cultured for 4 d in DMEM media (Invitrogen, cat. no. A1443001, Carlsbad, CA, USA) supplemented with 10% FBS containing either high glucose (4500 mg L^–1^) and high glutamine (2 mM) (H/H) or low glucose (450 mg/L) and low glutamine (0.5 mM) (L/L), respectively. All cell lines were confirmed to be mycoplasma-negative by PCR-based testing.

**Antibodies and reagents**

Antibodies against BRD4 [cat. no. 13440; western blotting, 1:1,000; IP/ChIP/CUT&RUN, 1 µL; immunohistochemistry (IHC, human tissue), 1:20,000], ATF3 (cat. no. 18665; western blotting, 1:1,000; IP/CUT&RUN, 1 µL), c-JUN (cat. no. 9165; western blotting, 1:1,000; IP/ CUT&RUN, 1 µL), p-AMPK (Thr172) (cat. no. 50081; western blotting, 1:1,000), phosphorylated ACC (Ser 79)(cat. no. 11818; western blotting, 1:1,000), CBP (cat. no. 7389; western blotting, 1:1,000), and P300 (cat. no. 86377; western blotting, 1:1,000) were sourced from Cell Signaling Technology (Danvers, MA, USA). ACC (cat. no. SAB4501395; western blotting, 1:1,000), anti-HA (cat. no. H9658; western blotting, 1:5,000) and anti-β-actin (cat. no. A1978; western blotting, 1:10,000) antibodies were acquired from Sigma-Aldrich (St. Louis, MO, USA). BRD4 (cat. no. Ab75898; IHC, mouse tissue, 1:200), EDN1 (cat. no. ab2786; IHC, 1:500; western blotting, 1:1,000) and CSF1 (cat. no. ab52864; IHC, 1:100) was acquired from Abcam (Cambridge, UK). jetPEI transfection reagent was purchased from Polyplus-transfection (Illkirch-Graffenstaden, France) and Lipofectamine 2000 transfection reagent was obtained from Invitrogen (Carlsbad, CA, USA). Glucose (cat. no. A2494001) and Glutamine (cat. no. 35050061) was from Invitrogen. MZ1 (cat. no. HY-107425) and JQ1 (cat. no. HY-13030) was purchased from MedChemExpress (Monmouth, NJ, USA). Digitonin (cat. no. D141-100MG), Concanavalin A (cat. no. C2272-2MG), and 4-Thiouridine (4-SU) (cat. no. T4509) was from Sigma-Aldrich. Dynabeads™ MyOne™ Streptavidin T1/C1 (cat. no. 65801D) was from Invitrogen.

The Duolink® In Situ Red Starter Kit Mouse/Rabbit (cat no. DUO92101-1KT) used for proximity ligation assay was obtained from sigma.

**Liquid chromatography–mass spectrometry (LC–MS) analysis of glucose and glutamine concentration in xenograft tumor**

Glucose and glutamine were extracted from tumor tissues as previously described.[1, 2] Briefly, mice were euthanized by cervical dislocation, and tumors were harvested and immediately placed on ice. Tissues were flash-frozen in liquid nitrogen before peripheral and core regions were isolated on ice. Immediately after isolation, peripheral and core tissues were immersed in 250~500 µL pre-cooled 80% methanol (−80 °C) prepared in HPLC-grade water and homogenized using a mortar and pestle on ice. The homogenate was centrifuged at 4 °C, and the supernatant was transferred to a new 1.5 mL tube for subsequent analysis using a SCIEX 4500 QTRAP mass spectrometer and Shimadzu 40 LC system. The LC–MS facility reported metabolite concentrations in µg/mL, which were converted to µmol/g tissue using the following formula:

The LC–MS data are given in **Table S1**.

**Establishment of CRISPR/Cas9 edited cells and molecular cloning**

Gene knockouts (KOs) and enhancer deleted cells were generated using the CRISPR/Cas9 system with guide RNAs (gRNAs) targeting specific genomic sequences. gRNAs were designed using the CRISPick portal of the Broad Institute and cloned into the BbsI site of the eSpCas9(1.1)-T2A-puro vector (cat. no. 101039, Addgene; donated by Andrea Nemeth). Cells were transfected using Lipofectamine 3000, followed by selection with 2 μg mL^–1^ puromycin for 48 h to establish stable KO cell lines. Surviving cells were pooled and expanded without single-cell cloning. For the generation of EDN1 enhancer-deleted monoclonal cell lines, puromycin-selected cells were seeded into 96-well plates at a density of 0.5 cell per 100 µL of culture medium per well by limiting dilution. Single-cell-derived colonies were allowed to expand for approximately 2 weeks. Genomic DNA was prepared using DirectPCR Cell Lysis Reagent (Viagen Biotech, Los Angeles, CA, USA), and deletion of enhancer was confirmed by PCR. For CRISPR activation (CRISPRa), the vector was constructed using Gibson assembly. A transposon vector containing PiggyBac inverted terminal repeats was obtained from VectorBuilder (cat. no. VB250929-1472het). The catalytic domain of human p300 (aa 1048–1664) was amplified from HEK293 cDNA. dCas9 was amplified from pAAVS1-NDi-CRISPRi (Addgene, plasmid # 73498). Both dCas9 and P300 were cloned into linearized transposon vectors via Gibson assembly. The final plasmid construct was verified by nanopore sequencing. KO efficiency was validated by western blotting, whereas CRISPRa activity was confirmed by qPCR analysis of target gene expression and by CUT&RUN assay. Primer sequences used in this study are listed in **Table S2.**

**Protein immunoblotting and immunoprecipitation**

Cell pellets were lysed in RIPA buffer (150 mM NaCl; 50 mM Tris-HCl, pH 7.4; 0.25% sodium deoxycholate; 1 mM EDTA; 1% NP-40; 1 mM NaF; 0.2 mM phenylmethylsulphonyl fluoride; 0.1 mM sodium orthovanadate) supplemented with protease/phosphatase inhibitor cocktails. Protein samples (10–30 µg) were separated using sodium dodecyl sulfate-polyacrylamide agarose gel electrophoresis, transferred onto polyvinylidene fluoride membranes (Merck Millipore, Burlington, MA, USA), and probed with specific antibodies. Co-immunoprecipitation involved cell harvesting and lysis using NET-NL buffer (50 mM Tris-HCl, pH 7.5; 150 mM NaCl; 0.5% NP-40; 1 mM EDTA; 1 mM dithiothreitol; 0.2 mM phenylmethylsulphonyl fluoride) supplemented with protease and phosphatase inhibitor cocktails. The lysate was subjected to immunoprecipitation with the indicated antibodies, followed by immunoblotting. Immunoblots were visualized using SuperSignal West Femto chemiluminescence substrate (cat. no. 34094, Invitrogen) and an Amersham Imager 680 (GE Healthcare, Chicago, IL, USA).

**Enzyme linked Immunosorbent assay for ATP measurement**

ATP measurement was done with EZ-ATP Assay Kit (cat. no. DG-ATP100, DoGen Bio, Seoul, Republic of Korea). Briefly, MCF7 (H/H) and MCF7 (L/L) cells were lysed in ice-cold RIPA buffer. Following centrifugation at 12,000 × g for 10 min, cell lysates containing 50 µg of protein were used for ATP measurement according to the manufacturer’s instructions.

**3-(4,5-Dimethylthiazol-2-yl)-2,5-diphenyltetrazolium bromide assay**

Cell viability was determined using an EZ-Cytox Cell viability assay kit (Daeli Lab Service, Seoul, Republic of Korea). Briefly, 2,500 MCF7 (H/H) and MCF7 (L/L) cells were seeded in 96-well plates with 100 μL cell suspension in each well and incubated at 37 °C in humidified air at the indicated time points. The cells were treated with 10 μL tetrazolium salt and incubated for 4 h. The absorbance of purple formazan formed by live cells was measured at 450 nm using a microplate reader (Molecular Devices, San Jose, CA).

**BrdU incorporation assay**

BrdU incorporation was measured using Cell Proliferation ELISA kit (cat no. 11647229001 Roche (Basel, Switzerland). Briefly, 2,500 MCF7 (H/H) and MCF7 (L/L) cells were seeded into 96-well plates in 100 μL of L/L medium per well and incubated at 37 °C in humidified air for the indicated time periods. Cells were treated with BrdU reagent for 4 h, and the remaining procedures were performed according to the manufacturer’s instructions.

**Cell cycle assay**

MCF7 (H/H) and MCF7 (L/L) cells were grown in L/L media at the indicated time points. The cells were washed, fixed with 70% ethanol, and treated with 200 μL Muse Cell cycle reagent (Merck Millipore). After the cells were incubated at 25 °C for 30 min in the dark, the cell cycle was analyzed using a Muse Cell Cycle Assay kit (Merck Millipore).

**RNA-seq**

Total RNA from MCF7 (H/H) and MCF7 (L/L) cells was extracted using the AccuPrep® Universal RNA Extraction Kit (Bioneer, Daejeon, South Korea). Briefly, 1 µg of total RNA was used for library preparation with the NEBNext Directional RNA Library Prep Kit (cat. no E7760S, New England Biolabs, Ipswich, MA, USA) in combination with the NEBNext Poly(A) mRNA Magnetic Isolation Module (cat. no. E7490S, New England Biolabs) according to the manufacturer’s instructions. The prepared libraries were sequenced on the NovaSeq 6000 platform by DNA Link (Seoul, South Korea).

**Reverse transcription (RT)-quantitative polymerase chain reaction (qPCR) and PCR**

Total RNA was treated with the TURBO DNA-free™ Kit (cat. no. AM1907, Invitrogen) to remove residual genomic DNA contamination. Briefly, 1 µg of total RNA was reverse-transcribed into cDNA using TOPscript™ RT DryMIX (dN6) (cat. no. RT210, Enzynomics, Daejeon, South Korea) according to the manufacturer’s instructions. The resulting cDNA was diluted 1:10 in nuclease-free water and used as the template for quantitative PCR (qPCR) using Fast SYBR Green qPCR PreMIX (cat. no. RT540S, Enzynomics) on a CFX Duet Real-Time PCR System (Bio-Rad, Hercules, CA, USA). For semi-quantitative PCR, AccuPower® GoldHotstart Taq PCR PreMix was used, and PCR products were resolved on a 1% agarose gel.

**Immunofluorescence microscopy**

MCF7 (H/H) and MCF7 (L/L) cells were seeded on coverslips, fixed using Cytofix/Cytoperm reagent (BD Biosciences, San Diego, CA), and then probed with anti-BRD4 antibody followed by either anti-mouse IgG-Alexa Fluor 647 antibody (Thermo Fisher Scientific) or anti-rabbit IgG-Alexa Flur 498 (Thermo Fisher Scientific). The coverslips were mounted on slides using ProLong Gold Antifade mountant with DAPI (Thermo Fisher Scientific) and examined using a confocal microscope (Nikon, Minato, Japan). The fluorescence intensity was measured using Fiji software (downloaded from https://fiji.sc/).

**Chromatin immunoprecipitation (ChIP)-quantitative polymerase chain reaction (qPCR) and ChIP-Seq**

ChIP analysis was performed using an EZ-Magna ChIP A/G Chromatin Immunoprecipitation Kit (Sigma-Aldrich) according to the manufacturer’s instructions, with the following modification: 1 ×107 cells were lysed in SDS lysis buffer and sonicated for 8 min (2 s on, 2 s off) at 40% amplitude using a probe sonicator (Vibra-Cell VCX 130). The remaining ChIP procedure were performed as recommended by manufacturers. The ChIP eluate (5 µl) was diluted at 1:10 in nuclease-free water and amplified via the indicated primers targeting enhancer regions. The PCR products were separated through AGE and visualized using a Davinci-gel imaging system (Davinci-K, Seoul, Republic of Korea) or used for qPCR. The Ct-values for ChIP samples were normalized to 10% input sample. Relative fold-change was calculated using the 2^–ΔΔCt^ method. The remaining eluate (~45 µL) was used for NGS library preparation using a NEBNext Ultra II DNA Library Prep Kit for Illumina (cat. no. E7645S, New England Biolabs) according to the manufacturer’s protocol. The prepared libraries were sequenced on the NovaSeq 6000 platform by DNA Link (Seoul, South Korea).

**CUT&RUN assay and CUT&RUN-qPCR**

CUT&RUN assay was performed as previously described with few modifications.[3] Briefly, 5 × 10^5^ cells were attached to concanavalin A‑coated magnetic beads prepared as previously described.[4] Cells were permeabilized with antibody buffer (20 mM HEPES, pH 7.5; 150 mM NaCl; 0.5 mM spermidine; 0.05% digitonin; 2 mM EDTA) and probed with 1 µL of the indicated antibodies in this buffer at 4 °C overnight without rotation. Cells were washed three times with Dig‑wash buffer (20 mM HEPES, pH 7.5; 150 mM NaCl, 0.5 mM spermidine, 0.05% digitonin) and then incubated with 1.5 µL Protein A/G‑MNase (cat. no. 40366, Cell Signaling Technology) in a 50 µL volume for 3 h at 4 °C. After three additional washes with Dig‑wash buffer, MNase was activated by the addition of 2 mM CaCl_2_ for 30 min at 4 °C. The MNase reaction was stopped by adding equal volume of 2X STOP buffer (340 mM NaCl, 20 mM EDTA, 4 mM EGTA, 0.05% Digitonin, and 100 µg mL^–1^ RNase A) and the mixture was incubated at 37 °C for 30 min. DNA in the supernatant was purified using a GeneJET Gel Extraction and DNA Cleanup Micro Kit (Thermo Fisher Scientific) and used as template for qPCR or PCR experiments. For input samples, genomic DNA was extracted from 5 × 10^5^ cells using an AccuPrep Genomic DNA Extraction Kit (Bioneer). The Ct-value for CUT&RUN samples was normalized to genomic DNA input. Relative fold-change between two or more groups was calculated using the 2^–ΔΔCt^ method.

**Proximity ligation assay (PLA)**

The PLA was performed using a Duolink In Situ Red Starter Kit Mouse/Rabbit, according to the manufacturer's instructions (Sigma). Briefly, cells were seeded onto 12-well chamber slides (SPL Life Sciences, #304012), fixed with 4% formaldehyde at room temperature for 15 min, and washed three times with PBS. Cells were then permeabilized with 0.05% Triton X-100 in PBS for 20 min at room temperature. Blocking was performed using the provided blocking buffer. Cells were incubated with antibodies against BRD4 (1:1,600) and ATF3/JUN (1:100) for 14 h at 4°C. All subsequent steps were performed according to the manufacturer’s instructions. Immunofluorescence were visualized under confocal microscope (Nikon Corporation, Tokyo, Japan)

**Transient transcriptome sequencing (TT-seq)**

TT‑seq was performed as previously described, with modifications.[5] Briefly, MCF7 (H/H) and MCF7 (L/L) cells were treated with 700 µM 4‑thiouridine (4‑SU) for 10 min. Total RNA was extracted using TRIzol reagent and dissolved in nuclease‑free water. A total of 160 µg RNA was mixed with 40 µL 10× biotinylation buffer (100 mM HEPES–KOH, pH 7.5; 10 mM EDTA) and 0.05% spike‑in RNA cocktail, adjusting the final volume to 400 µL with nuclease‑free water; the final RNA concentration in the biotinylation mix was 0.4 µg µL^–1^. This solution was mixed with 100 µL MTSEA‑XX‑Biotin (166 µg mL^–1^ dissolved in DMF) and incubated for 4 h at room temperature in amber tubes with continuous rotation. RNA was then purified through standard isopropanol precipitation and resuspended in nuclease‑free water. RNA was fragmented using fragmentation buffer (10 mM Tris–HCl, pH 7.5; 10 mM ZnCl_2_) and enriched according as previously described.[5] 2-4 µL eluate was used for dot blotting and qPCR to check the efficiency of biotinylation and spike-in RNA enrichment. The sequencing library was prepared using a NEBNext Directional RNA Library Prep Kit according to the manufacturer’s instructions. The prepared libraries were sequenced on the NovaSeq X platform by Macrogen (Seoul, South Korea).

**Spike‑in RNA synthesis for TT‑seq**

The spike‑in RNA comprised a 1:1 mixture of Firefly luciferase (Fluc) RNA and Renilla luciferase (Rluc) RNA. Both spike‑in RNAs were synthesized using an EZ T7 High Yield In Vitro Transcription Kit (Enzynomics, Daejeon, Republic of Korea) from a psiCHECK‑3 plasmid template (cat. no. 136010, Addgene, MA, USA). Fluc RNA was transcribed using the recommended NTP concentrations, whereas Rluc RNA was synthesized in the presence of 10% 4SU‑UTP (cat. no. NU-1156S, Jena Bioscience, Jena, Germany) supplemented with UTP, such that approximately 1/10 uridine bases is replaced by 4‑thiouridine. After transcription, template plasmid DNA was removed using a TURBO DNA‑free Kit (Thermo Fisher Scientific).

**Antisense oligonucleotide transfection**

Gapmer-type antisense oligonucleotides (ASOs) were synthesized by Integrated DNA Technologies (IDT). Four distinct ASOs were pooled and transfected using Lipofectamine 2000 reagent, with each ASO at a final concentration of 10 nM (total ASO concentration: 40 nM). For the control group, a non-targeting control ASO was transfected at a final concentration of 40 nM. The ASO

**Wound healing assay**

A wound healing assay was performed using a Culture‑Insert 2 Well (ibidi GmbH, Gräfelfing, Germany) placed in a µ‑Dish 35 mm. Cells were seeded in 60 mm dishes and cultured in either H/H or L/L medium for 4 d. For experiments involving ASO transfection, ASOs were transfected 48 h before cell harvesting during the culture period. Cells were harvested, resuspended in either H/H or L/L media, and adjusted to a concentration of ~3-5 × 10⁵ cells mL^–1^ to yield a confluent monolayer after 24 h. A volume of 70 µL cell suspension was added into each well of the Culture‑Insert, and the dish was incubated at 37 °C in a humidified atmosphere with 5% CO_2_ for at least 24 h to allow cell attachment and confluence.

After attachment, the insert was gently removed with sterile tweezers, generating a defined cell‑free gap of ~500 µm. The cell monolayer was washed once with PBS or fresh medium to remove detached cells and debris, followed by the addition of fresh H/H or L/L medium containing the desired concentration of MZ1. Images of the gap were acquired after 24 h for MDA-MB-231 and after 72 h for MCF7 cells. The wound closure area was quantified from the images using ImageJ (version 1.5.3t, NIH, Bethesda, MD, USA), and the relative gap closure over time was calculated as a measure of cell migration.

**Matrigel cell invasion assay**

A Matrigel invasion assay was performed using transwell inserts coated with Matrigel basement‑membrane matrix (cat. no. CLS356234; Corning, NY, USA). MCF (H/H) and MCF7 (L/L) cells were seeded in serum‑free L/L medium in the upper chamber, and medium containing chemoattractant (i.e., 10% FBS) was placed in the lower chamber. After incubation for 72 h, non‑invading cells on the upper surface were removed using a cotton swab and cells that had invaded through the Matrigel to the lower membrane were stained with DAPI for image acquisition.

**Orthotopic mouse model**

Female BALB/c mice (6 weeks old) were purchased from Orient Bio Inc. (Seongnam, Republic of Korea) and housed in separate cages under controlled light and temperature conditions. They were provided commercial rat chow (Orient Bio Inc.) and had unrestricted access to water, in accordance with standard housing guidelines for mice. Mice were randomly allocated using block randomization method to three groups, with the exact number of animals per group specified for each experiment. 4T1-Luc cells (5 × 10^5^ cells in 100 µL PBS) were injected into the abdominal mammary fat pads of mice and allowed to grow for 1 week. Bioluminescence images corresponding to the pre-treatment baseline were acquired at this time point. Tumors were then allowed to grow for an additional ~2 weeks until they reached an approximate volume of 300 mm^3^, after which drug treatment was initiated.

For drug administration, compounds were initially dissolved in DMSO and subsequently prepared as a 10 mg kg^–1^ formulation containing 10% Tween-80 and 10% DMSO in PBS. JQ1 and MZ1 were administered via intraperitoneal injection three times per week for 2 weeks. Tumor size was measured using Vernier calipers three times per week, concomitant with the drug treatment schedule. Tumor volume was calculated as follows: Volume = 0.5 × [(largest diameter) × (smallest diameter)^2^]. One week after cessation of drug treatment, bioluminescence images were acquired using the IVIS NightOWL system (PerkinElmer, Shelton, CT, USA) following intraperitoneal injection of 150 mg kg^–1^ D-Luciferin (cat. no. HY-12591A, MedChemExpress). At the end of the experiment, mice were euthanized by cervical dislocation, and the tumors and lungs were excised for subsequent analyses. The study protocol was approved by the Animal Experiments Committee of Chosun University (CIACUC 2025-S0037).

**Mouse xenograft model**

Female BALB/c nude mice (4 weeks old) were obtained from Orient Bio and maintained in separate cages under controlled temperature and light conditions. After 1 week of acclimation, CRISPRa cells were subcutaneously injected into the flanks of the mice (1 × 10^6^ cells suspended in 50 µL ice-cold PBS mixed with 50 µL Matrigel basement membrane matrix; total volume, 100 µL), and tumors were allowed to grow for 1 month. 17β-Estradiol (3 mg kg^–1^), dissolved in a 10% Tween-80 solution in PBS, was administered every other day beginning 2 days prior to cell implantation and continued throughout the experimental period. MZ1 (10 mg kg^–1^) was administered via intraperitoneal injection five times per week for 2 weeks. Following an additional 2 week interval after completion of drug treatment, mice were euthanized by cervical dislocation and tumors were harvested.

For antisense oligonucleotide (ASO) treatment, the same procedure described above was followed, except that parental MCF7 cells were implanted instead of MCF7-CRISPRa cells. ASOs were administered via intratumoral injection at a dose of 1 µg/mouse after complex formation with jetPEI (2 µL PEI per µg ASO cocktail) in a final volume of 10 µL. Treatments were administered three times per week for 2 weeks. Following an additional 2 week interval after completion of treatment, mice were euthanized and tumors were harvested. The tumor diameter was measured using calipers (Mitutoyo, Kawasaki, Japan) and tumor volumes were calculated as described for the orthotopic mouse model. The study was conducted according to the ARRIVE guidelines and approved by the Animal Experiments Committee of Chosun University (CIACUC 2025-S0037).

For LC–MS measurements of intratumoral glucose and glutamine, the same procedure was followed as for xenograft tumors, except that 5 × 10^6^ MCF7 cells were implanted, and tumors were allowed to grow for 1 month before collection for metabolite analysis.

**Immunohistochemistry**

Informed consent was obtained from all participants, and the study was conducted following protocols approved by the Ethics Committee of Chosun University Hospital in accordance with the Declaration of Helsinki. Tissue samples (45 cases) were collected from 30 patients diagnosed with invasive breast carcinoma, aged between 42 and 72 years. IHC was conducted according to previously established methods.[6] Each specimen was evaluated based on the percentage of cells exhibiting positive nuclear/cytoplasmic staining (score of 1–5) and intensity of staining (score of 1–3). The final IHC score for each sample was determined by multiplying the percentage and intensity scores.

**Bioinformatics data analysis**

For RNA-seq data analysis, fastq files were processed using fastp (version 1.3.3). Processed reads were aligned to the human reference genome GRCh38 assembly using Salmon (version 1.10.3, available at <https://github.com/COMBINE-lab/salmon>, last accessed: April 22, 2026) The salmon index was obtained from refgenie (available at refgenome.databio.org). The salmon output was imported into R using tximeta (version 3.22). Differential gene expression was analyzed using edgeR (version 4.6.3). Gene Ontology enrichment analysis was performed using clusterProfiler (version 4.6.10). Gene set enrichment analysis was performed with GSEA (version 4.4.0)

For ChIP-seq data analysis, fastq files were processed using fastp (version 1.3.3). Processed reads were aligned to the human reference genome GRCh38 assembly using bowtie2 (version 2.5.4, available at <https://bowtie-bio.sourceforge.net/bowtie2/index.shtml>). The reference index for bowtie was downloaded from the home repository given above. The heatmap and coverage plots were prepared using deeptools (version 3.5.6). The bigwig file was generated using deeptools with the following options: --normalizeUsing RPKM -bl ENCFF356LFX.bed. Default options were used for other arguments. The bed file containing the blacklisted region was obtained from ENCODE (available at <https://www.encodeproject.org/annotations/ENCSR636HFF/>). For peak calling with replicates, Binary Alignment/Map (BAM) files were merged into a single BAM file and run in MACS2 (version 3.0.3) with the following options: -t ChIP.bam -c Input.bam -f BAMPE, -q 0.05. However, for MCF7 (L/L)-Unique peaks, MACS2 was run using the following options: -t LL.bam -c HH.bam -f BAME -q 0.05. Default options and arguments were used for other parameters. The enhancer regions were called using Rank Ordering of Super-Enhancers (version 1.3.2, available at <https://github.com/stjude/ROSE>). The overlap of H3K27ac peaks (**Figure 2b**) was analyzed using GenomicRanges (version 1.56.2, available at Bioconductor.org). The distribution of ChIP peaks was analyzed using ChIPseeker (version 1.40.0, available at Bioconductor.org).

For TT-seq analysis, ~40 million paired (150 PE) sequences were generated via Illumina sequencing. Fastq files were processed using fastp (version 1.3.3) with default parameters. Processed reads were aligned to the human reference genome GRCh38 assembly using STAR (version 2.7.11b, available at https://github.com/alexdobin/STAR) using default settings with –quantMode GeneCounts as an additional option. The overall mapping rate for all samples was >90%. Subsequently, 35–37 million uniquely mapped reads were available for further analysis. Count information available in the ReadsPerGene.out.tab file produced by STAR was directly imported in R and differential gene expression and Gene Ontology analyses were performed using edgeR and clusterProfiler (**Figure 4b–d**). Enhancer RNA (eRNA) identification was performed as previously described (**Figure S4e**).[5] Briefly, BAM files from MCF7 (H/H) and MCF7 (L/L) cells were merged, and de novo transcript assembly was performed using StringTie (version 3.0.3) without a reference GTF. Transcripts overlapping GENCODE version 49-annotated transcripts on the same strand were removed, except for lncRNAs. Transcripts overlapping upstream antisense RNAs, defined as transcripts originating from the −300 bp to +1000 bp region relative to the transcription start site (TSS) of protein-coding genes on the opposite strand, were further excluded. The remaining transcripts were exported as an eRNA.gtf file. Reads mapping to eRNA.gtf were summarized using featureCounts with the options -p, -t transcript, and -s 2. Count matrices were imported into R for differential expression analysis using edgeR. To enrich for active eRNAs, only transcripts whose TSS overlapped H3K27ac peaks were retained using the promoters function in GenomicRanges and subsetByOverlaps in IRanges. Differentially expressed eRNAs were visualized using EnhancedVolcano (**Figure 4e**) the cut-off values are indicated within corresponding figure legend.

**Statistical analysis**

Statistical analyses were performed using Prism 8 (GraphPad Software, San Diego, CA, USA). A two-tailed Student’s *t*-test was employed for comparing two independent groups, while one-way analysis of variance followed by Tukey’s multiple comparisons test or two-way analysis of variance followed by Dunnett’s or Sidak’s multiple comparison test was used for multiple comparison analyses. Statistical significance was set at *p* < 0.05.

**References**

1.	Pan, M.; Reid, M. A.; Lowman, X. H.; Kulkarni, R. P.; Tran, T. Q.; Liu, X.; Yang, Y.; Hernandez-Davies, J. E.; Rosales, K. K.; Li, H.; Hugo, W.; Song, C.; Xu, X.; Schones, D. E.; Ann, D. K.; Gradinaru, V.; Lo, R. S.; Locasale, J. W.; Kong, M., Regional glutamine deficiency in tumours promotes dedifferentiation through inhibition of histone demethylation. *Nat Cell Biol* **2016,** 18, (10), 1090-101.

2.	Reid, M. A.; Wang, W. I.; Rosales, K. R.; Welliver, M. X.; Pan, M.; Kong, M., The B55α subunit of PP2A drives a p53-dependent metabolic adaptation to glutamine deprivation. *Mol Cell* **2013,** 50, (2), 200-11.

3.	Meers, M. P.; Bryson, T. D.; Henikoff, J. G.; Henikoff, S., Improved CUT&RUN chromatin profiling tools. *eLife* **2019,** 8, e46314.

4.	Fujiwara, Y.; Tanno, Y.; Sugishita, H.; Kishi, Y.; Makino, Y.; Okada, Y., Preparation of optimized concanavalin A-conjugated Dynabeads® magnetic beads for CUT&Tag. *PLOS ONE* **2021,** 16, (11), e0259846.

5.	Lee, J.-H.; Wang, R.; Xiong, F.; Krakowiak, J.; Liao, Z.; Nguyen, P. T.; Moroz-Omori, E. V.; Shao, J.; Zhu, X.; Bolt, M. J.; Wu, H.; Singh, P. K.; Bi, M.; Shi, C. J.; Jamal, N.; Li, G.; Mistry, R.; Jung, S. Y.; Tsai, K.-L.; Ferreon, J. C.; Stossi, F.; Caflisch, A.; Liu, Z.; Mancini, M. A.; Li, W., Enhancer RNA m6A methylation facilitates transcriptional condensate formation and gene activation. *Molecular Cell* **2021,** 81, (16), 3368-3385.e9.

6.	Choe, J.; Lin, S.; Zhang, W.; Liu, Q.; Wang, L.; Ramirez-Moya, J.; Du, P.; Kim, W.; Tang, S.; Sliz, P.; Santisteban, P.; George, R. E.; Richards, W. G.; Wong, K. K.; Locker, N.; Slack, F. J.; Gregory, R. I., mRNA circularization by METTL3-eIF3h enhances translation and promotes oncogenesis. *Nature* **2018,** 561, (7724), 556-560.

**Figure S1. Characterization of nutrient deprivation-induced phenotypic and transcriptional changes in breast cancer cells.**

a) Bar plot of different amino acids and α-ketoglutarate (α-KG) levels, expressed as core/periphery ratio, in xenograft tumors formed by M229 cells. Glutamine and its metabolic products are shown in red, from Pan et al (see Supplementary Table 1) [1]. The inset shows a glutamine metabolism pathway.

b) Cell morphology of MCF7 and MDA-MB-231 cells upon culture in H/H and L/L media for 4 d.

c) Western blot analysis of MDA-MB-231 (H/H) and MDA-MB-231 (L/L) cells probed with the indicated antibodies.

d) Relative proliferation of MCF7 cells cultured in H/H or L/L medium assessed using 3-(4,5-dimethylthiazol-2-yl)-2,5-diphenyltetrazolium bromide assay. Data are presented as mean ± SD (*n* = 3). ****p* < 0.001 (Student’s *t*-test; compared with H/H at each time point).

e) Relative BrdU incorporation of MCF7 (H/H) and MCF7 (L/L) cells in L/L medium assessed using BrdU incorporation assay. Data are presented as mean ± SD (*n* = 3). ****p* < 0.001 (Student’s *t*-test; compared with MCF7 (H/H) at each time point).

f) Dot plot showing the biological coefficient of variation (BCV) between MCF7 (H/H) and MCF7 (L/L) RNA-seq libraries produced using edgeR.

g) Plot showing the clustering of RNA-seq libraries using principal component analysis.

h) KEGG pathways associated with genes significantly overexpressed in MCF7 (L/L) cells, ranked in descending order of normalized enrichment score (NES). An FDR q-value of 0 indicates an extremely significant pathway rather than a calculation error. This value arises from the statistical reporting and numerical precision of the GSEA algorithm.

**Figure S2. Identification and functional validation of nutrient-responsive BRD4-associated enhancers in breast cancer cells.**

a) H3K27ac density between MCF7 (H/H) and MCF7 (L/L) across transcription start site (TSS) and transcription end site (TES).

b) Hockey stick plots showing the ranking of enhancers and super‑enhancers in MCF7 (H/H), MCF7 (L/L), and MCF7 (L/L)‑unique H3K27ac peaks based on ROSE analysis. The numbers of typical enhancers and super‑enhancers are indicated within each panel.

c) Dot plot showing log₂ fold changes in H3K27ac signal at ROSE-identified enhancer regions and the RNA expression of their mapped genes. Dot size represents the product of the fold changes in H3K27ac coverage and gene expression, indicating the combined magnitude of enhancer activation and transcriptional response. Duplicated genes are removed for simplified view.

d) RT-qPCR with primers targeting indicated genes in MDA-MB-231 (H/H) and MDA-MB-231 (L/L) cells. Data are presented as mean ± SD (*n* = 3). ****p* < 0.001 (Student’s *t-*test; compared with H/H).

e) Western blot analysis of indicated enhancer-associated proteins in MDA-MB-231 (H/H) and MDA-MB-231 (L/L) cells.

f) ChIP‑qPCR showing BRD4 enrichment at the indicated enhancer regions in MCF7 (H/H) and MCF7 (L/L) cells. Data are presented as mean ± SD (*n* = 3). ****p* < 0.001 (Student’s *t*-test; compared with the corresponding H/H group in the BRD4 ChIP samples).

g) CUT&RUN–qPCR showing p300 enrichment at the indicated enhancer regions in MCF7 (H/H) and MCF7 (L/L) cells. Data are presented as mean ± SD (*n* = 3). ****p* < 0.001 (Student’s *t-*test; compared with the corresponding H/H group in the p300 ChIP samples).

h) Integrative Genomics Viewer (IGV) tracks showing H3K27ac coverage at the *EDN1* enhancer region in MCF7 (H/H) and MCF7 (L/L) cells. The binding sites of guide RNAs used for CRISPR-mediated enhancer deletion and PCR primers used to validate enhancer deletion are indicated. The guide RNA and primer sequence are provided in Table S2.

i) PCR analysis of *EDN1* enhancer using genomic DNA extracted from independent *EDN1* enhancer-deleted MCF7 cell clones (MCF7^ΔEDN1-enh^) and wild-type MCF7 cells (MCF7^WT^). PCR amplification of the *GAPDH* promoter region was used as a control. Clone #1 of MCF7^ΔEDN1-enh^ was used for further experiments.

j) RT-qPCR of EDN1 in MCF7^WT^ and MCF7^ΔEDN1-enh^ cells after culture in either H/H or L/L media for 4 days. Data are presented as mean ± SD (n = 3). ****p* < 0.001, ^n.s^ not significant (Student’s *t*-test; comparisons were made with the corresponding H/H group within each condition)

k, l) RT-PCR (k) and RT-qPCR (l) of MCF7 (H/H) and MCF7 (L/L) cells treated with MZ1 for the indicated durations using primers targeting the indicated genes, and detection of protein levels using western blotting in cell lysates under the corresponding conditions with the indicated antibodies. Data are presented as mean ± SD (n = 3). ***p* < 0.005, **p* < 0.05 (Student’s *t*-test; compared with H/H at corresponding time point).

**Figure S3. Transcription factor recruitment and interaction with BRD4 at nutrient-responsive enhancer regions.**

a) RT-qPCR with primers targeting indicated transcription factors in MDA-MB-231 (H/H) and MDA-MB-231 (L/L) cells. Data are presented as mean ± SD (*n* = 3). ****p* < 0.001, ***p* , 0.005 [Student’s *t*-test, compared with MDA-MB-231 (H/H)].

b) Western blot analysis of MDA-MB-231 (H/H) and MDA-MB-231 (L/L) cells using antibodies targeting the indicated proteins.

c) CUT&RUN–PCR showing enrichment of ATF3 and JUN at the enhancer regions of the indicated genes in MCF7 (H/H) and MCF7 (L/L) cells.

d) CUT&RUN–qPCR showing enrichment of ATF3 (left) and JUN (right) at the enhancer regions of the indicated genes in MCF7 (H/H) and MCF7 (L/L) cells. Data are presented as mean ± SD (*n* = 3). ****p* < 0.001, ***p* < 0.005 (Student’s *t*-test, comparisons were made with the corresponding H/H group for each ATF3 or JUN antibody)

e) Immunofluorescence imaging of in situ PLA signal (red) produced by probes targeting anti-ATF3/JUN (raised in rabbit) and anti-BRD4 (raised in mouse) in MCF7 (H/H) and MCF7 (L/L) cells. Cell nuclei are counterstained with 4′,6-diamidino-2-phenylindole (blue).

**Figure S4. Functional validation of enhancer-derived eRNAs using antisense oligonucleotide-mediated depletion.**

a) Dot blot assay showing 4‑SU incorporation into nascent RNA, as detected by biotinylation of 4‑SU with biotin‑MTSEA and subsequent visualization using streptavidin‑HRP.

b) RT‑qPCR showing enrichment of Fluc and Rluc spike‑in RNAs following affinity enrichment of biotinylated nascent RNA from MCF7 (H/H) and MCF7 (L/L) cells. Data are presented as mean ± SD (*n* = 3). ****p* < 0.001 (Student’s *t-*test; compared with Fluc).

c) Principal component analysis (PCA) plot showing the clustering of TT-seq libraries.

d) Dot plot showing the BCV between H/H and L/L TT-seq libraries analyzed using edgeR.

e) Schematic illustration of data analysis workflow used to identify eRNAs from TT-seq data. uaRNA and TSS denote upstream antisense RNA and transcription start site, respectively.

f) RT-qPCR with primers targeting *EDN1e* eRNA in MDA-MB-231 (H/H) and MDA-MB-231 (L/L) cells. Data are presented as mean ± SD (*n* = 3). **p* < 0.05 (Student’s *t-*test; compared with H/H).

g) IGV tracks showing TT-seq and H3K27ac ChIP-seq coverage across the *EDN1* enhancer, with the ASO target site indicated. The data range is indicated at the top of the y-axis.

h) RT-qPCR of MCF7 cells transfected with ASO-Ctrl or independent ASO-EDN1e and cultured in L/L medium for 4 d with primers targeting *EDN1e* eRNA. Data are presented as mean ± SD (*n* = 3). **p* < 0.05 (one-way ANOVA, compared with Ctrl in each group).

i) RT-qPCR of MCF7^WT^ and MCF7^ΔEDN1-enh^ cells transfected with ASO-Ctrl or ASO-EDN1e and cultured in L/L media for 4 d with primers targeting EDN1 mRNA. Data are presented as mean ± SD (*n* = 3). ***p* < 0.005, ^n.s.^ not significant (one-way ANOVA; compared with Ctrl in each group).

j) CUT&RUN–qPCR showing p300 enrichment at *EDN1* enhancer in MCF7 cells transfected with ASO-Ctrl or ASO-EDN1e and cultured in H/H or L/L medium for 4 d. IgG serves as negative control. Data are presented as mean ± SD (*n* = 3). ***^, ###^*p* < 0.001 (one-way ANOVA; *** compared with H/H-Ctrl-p300; ### compared with L/L-Ctrl-p300).

k) RT-qPCR of MDA-MB-231 cells transfected with ASO-Ctrl or ASO-EDN1e and cultured in H/H or L/L medium for 4 d with primers targeting *EDN1* mRNA. Data are presented as mean ± SD (*n* = 3). ***^, ###^*p* < 0.001 (one-way ANOVA; *** compared with H/H-Ctrl; ### compared with L/L-Ctrl).

**Figure S5. Inhibition of nutrient deprivation-induced cell migration by targeting enhancer/eRNA activity.**

a) Representative images of wound‑healing assay with MDA-MB-231 (H/H) and MDA-MB-231 (L/L) cells treated with the indicated concentration of MZ1 for 24 h (left). Wound closure rate is expressed as the percentage of closure at 24 h relative to 0 h (right). Data are presented as mean ± SD (*n* = 3). ****p* < 0.001 [one-way ANOVA; compared with L/L-MZ1(-)]

b) RT-PCR analysis of MCF7 cells transfected with sgCtrl and sgEDN1 and cultured in H/H or L/L medium, using primers for the indicated genes. Protein expression in respective cell lysate was analyzed with western blotting using indicated antibodies.

c) RT-PCR analysis of MCF7 cells transfected with sgCtrl, sgCSF1, sgTGFB2, or sgVTN and cultured in H/H or L/L medium, using primers for the indicated genes.

d) Representative images of wound-healing assay with MCF7 cells transfected with indicated sgRNAs and cultured in either H/H or L/L medium for 4 d (left). Wound closure rate is expressed as in panel (a) (right). Data are presented as mean ± SD (*n* = 3). ****p* < 0.001 (one-way ANOVA; compared with sgCtrl-L/L).

e) Representative images of wound‑healing assay with MDA-MB-231 cells transfected with ASO-Ctrl or ASO-EDN1e and cultured in either H/H or L/L medium for 4 d (left). Wound closure rate is expressed as in panel (a) (right). Data are presented as mean ± SD (*n* = 3). ****p* < 0.001 (one-way ANOVA; compared with Ctrl-L/L group).

f) Representative images of wound-healing assay with MCF7^WT^ and MCF7^ΔEDN1-enh^ cells cultured in either H/H or L/L medium for 4 d (left). Wound closure rate is expressed as in panel (a) (right). Data are presented as mean ± SD (*n* = 3). ****p* < 0.001 (one-way ANOVA; compared with MCF7^WT^-L/L group).

g) Representative images of three-dimensional spheroids derived from MCF7 (H/H) and MCF7 (L/L) cells and treated with increasing concentrations of MZ1 (left), along with quantified spheroid size (right). Data are presented as mean ± SD (*n* = 3), ****p* < 0.001, ***p* < 0.005 [one-way ANOVA; compared with the corresponding MCF7 (H/H) group at each concentration].

h) Representative images of tumors derived from 4T1-Luc cells treated with indicated drugs or PBS.

**Figure S6. Spatial activation of enhancer-dependent gene programs in nutrient-deprived tumor regions and their association with metastasis.**

a) Correlation between *BRD4* and *CSF1, TGFB2, and VTN* mRNA expression in Metastatic Breast Cancer Project dataset (2021, available from cbioportal.org). Pearson’s correlation (*r*) and significance (*P-*value) are indicated.

b) Representative IHC staining images of CSF1 in human breast cancer primary tumors (grade I–III) and lymph node metastases.

c) IHC scoring of CSF1 based on tumor progression and metastasis. Data are presented as mean ± SD (*n* = 10). **p* < 0.05 (one-way ANOVA; compared with Normal group).

d) RT-qPCR analyses of tumor samples obtained from peripheral and core tumor sections using indicated primers. Data are presented as mean ± SD (*n* = 3). ****p* < 0.001, ***p* < 0.005, ^n.s.^ not significant (Student’s *t*-test; compared with Periphery).

e) Western blot analysis of tumor lysates derived from peripheral and core tumor sections using indicated antibodies.

f, g) Western blot analysis of tumor lysates derived from MCF7 cells transfected with CRISPRa constructs (f) or treated with antisense oligonucleotides (g) using the indicated antibodies.

**Table S1. LC-MS Quantification of glucose and glutamine in MCF7 xenograft tumors**

| **Sample name** | | **Concentration (ug/ml)** | | **Tissue weight (g)** | **concentration (µmol/g)** | |
| --- | --- | --- | --- | --- | --- | --- |
|  |  | **Glutamine** | **Glucose** |  | **Glutamine** | **Glucose** |
| **Tumor core** | **1** | 3.19 | 2.43 | 0.0053 | 2.05914058 | 1.272454 |
|  | **2** | 3.57 | 2.56 | 0.0052 | 2.34874602 | 1.366307 |
|  | **3** | 3.84 | 1.52 | 0.0064 | 2.0526856 | 0.659136 |
|  | **4** | 3.32 | 2.5 | 0.002 | 2.83954841 | 1.734569 |
|  | **5** | 4.76 | 5.23 | 0.0047 | 1.73240841 | 1.544136 |
|  | **6** | 3.83 | 2.17 | 0.0032 | 2.04734006 | 0.941004 |
|  | **7** | 2.88 | 2.14 | 0.0021 | 2.3459264 | 1.414087 |
|  | **8** | 6.76 | 5.52 | 0.0049 | 2.35989024 | 1.563236 |
|  | **9** | 12 | 11.2 | 0.0089 | 2.30638831 | 1.746263 |
| **Tumor periphery** | **1** | 9.54 | 8.83 | 0.0138 | 2.3650508 | 1.775797 |
|  | **2** | 7.84 | 4.53 | 0.0062 | 4.32609008 | 2.027767 |
|  | **3** | 4.5 | 2.22 | 0.0063 | 2.44367333 | 0.977967 |
|  | **4** | 7.8 | 6.26 | 0.0041 | 3.25425765 | 2.118713 |
|  | **5** | 11.2 | 9.44 | 0.0062 | 3.09006434 | 2.112817 |
|  | **6** | 9.19 | 9.95 | 0.0021 | 7.48578597 | 6.574844 |
|  | **7** | 6.79 | 7.09 | 0.0035 | 3.31850838 | 2.810993 |
|  | **8** | 11.7 | 7.38 | 0.0044 | 4.54856468 | 2.327477 |
|  | **9** | 9.18 | 10.2 | 0.0051 | 3.0790284 | 2.775311 |
| Note: Samples 1–3 were extracted in 500 µL of 80% methanol, whereas Samples 4–9 were extracted in 250 µL of 80% methanol. | | | | | | |

**Table S2. List of oligonucleotide sequences used in this study**

| Oligo Name | Sequence | Purpose |
| --- | --- | --- |
| VTN_F | CCT CAA GAA CGG TTC CCT CT | RT-qPCR |
| VTN_R | CAT CCT CAA AGC GCC AGT AC |  |
| CSF1_F | TTC TCC ACT CCC TGC ATC AG |  |
| CSF1_R | GAT GAG ATC CTG GGA GAG CC |  |
| TGFB2_F | TGC TGC CTA CGT CCA CTT TA |  |
| TGFB2_R | GCT GTG CTG AGT GTC TGA AC |  |
| EDN1_F | TCT CTC TGC TGT TTG TGG CT |  |
| EDN1_R | CCA GGT GGC AGA AGT AGA CA |  |
| GAPDH_F | AAC TTT GGT ATC GTG GAA GGA |  |
| GAPDH_R | GAG GCA GGG ATG ATG TTCT |  |
| FLuc_F | TTC GCT AAG AGC ACC CTG AT |  |
| FLuc_R | GTA ATC AGA ATG GCG CTG GT |  |
| RLuc_F | GTA ACG CTG CCT CCA GCT AC |  |
| RLuc_R | CCA AGC GGT GAG GTA CTT GT |  |
| ATF3_F | TTG CCA TCC AGA ACA AGC AC |  |
| ATF3_R | GCA CTC CGT CTT CTC CTT CT |  |
| JUN_F | CAA GAA CTC GGA CCT CCT CA |  |
| JUN_R | TCC TGC TCA TCT GTC ACG TT |  |
| FOSB_F | GAA GAG GAG AAG CGA AGG GT |  |
| FOSB_R | CAC CAG CAC AAA CTC CAG AC |  |
| NPAS2_F | GGA GGT TTG CTT CAT TGC CA |  |
| NPAS2_R | CAG CAC TTC AAA AGG CAG GT |  |
| VTN-enh_F | AAT GAC GAA CCA TGA AGC GG | ChIP-qPCR/PCR and CUT&RUN-qPCR  Note: EDN1-enh primers were also used to verify EDN1-enh deletion. |
| VTN-enh_R | ACA GGC AAT TGA CAA GAG GA |  |
| CSF1-enh_F | AGC GCT TTG TAG GTA TCC GA |  |
| CSF1-enh_R | AGT CAC CCC TGC TCT ACA AC |  |
| TGFB2-enh_F | TGT CTG TCC CTT GCC TCA AA |  |
| TGFB2-enh_R | TAT TTC CCC ACA CTG TCC CC |  |
| EDN1-enh_F | GCT ACA GTG ATG CTC CTC CT |  |
| EDN1-enh_R | TGC ACA AAC TGA CTT CAC CG |  |
| CSF1e_F | GGG CAT TCT GGG GAG AGA G | RT-qPCR for enhancer RNA  Note: Unless explicitly stated otherwise, qPCR analysis of EDN1e was performed using primers specific for EDN1e(+). |
| CSF1e_R | GCT CCC TTT TGC CTG TTT CA |  |
| TGFB2e_F | GAG GAA GAG CTA GCT ACG CA |  |
| TGFB2e_R | TTT TCT GCT GTG TTG CCC TC |  |
| EDN1e(+)_F | AAC GCA CAA GGG ATG GTT TC |  |
| EDN1e(+)_R | GGG TAA GGG CTG GAT CCA AT |  |
| EDN1e(-)_F | ATGCCCACAAAGACACACAT |  |
| EDN1e(-)_R | GCTGATGTGAGTTGTTGGCT |  |
| sgEDN1 #1 | GA AGG AGC TCC AGA AAC AGG T | CRISPR/Cas9 knockout |
| sgEDN1 #2 | GG ACG AGC AGG AGC AGC GCT |  |
| sgJUN #1 | GC TGC CCA GCG TCA CGT CGG |  |
| sgJUN #2 | GG TCC GAG TTC TTG GCG CGG |  |
| sgATF3#1 | GT TGC TTG TCC CCC AGG AGT |  |
| sgATF3#2 | GG TGT CCA TCA CAA AAG CCG |  |
| sgEDN1-enh#1 | GA GGT ACA CCA CGA TGA ACA T | CRISPRa guide RNA  (Note: sgEDN1-enh#1 and sgEDN1-enh#2 were repurposed for EDN1 enhancer deletion using eSpCas9) |
| sgEDN1-enh#2 | GT CCT GAG GCT CTT ACA CTA |  |
| sgEDN1-enh#3 | GA AGA GGA AAA ACT CGA TAT C |  |
| sgTGFB2-enh#1 | GA TAG TTT ACT CAG ACT GAT G |  |
| sgTGFB2-enh#2 | GA AAC ATA GCC CGA AAA ATG |  |
| sgTGFB2-enh#3 | GA AGT ATG AGT GTC AGA TGC T |  |
| sgCSF1-enh#1 | CA TGT GAT AAG CCA GGC CCA |  |
| sgCSF1-enh#2 | TG TGT GTT CCC ACC ACA GGG |  |
| sgEDN1-enh#1 | GA GGT ACA CCA CGA TGA ACA T |  |
| sgEDN1-enh#2 | GT CCT GAG GCT CTT ACA CTA |  |
| sgEDN1-enh#3 | GA AGA GGA AAA ACT CGA TAT C |  |
| sgTGFB2-enh#1 | GA TAG TTT ACT CAG ACT GAT G |  |
| sgTGFB2-enh#2 | GA AAC ATA GCC CGA AAA ATG |  |
| sgTGFB2-enh#3 | GA AGT ATG AGT GTC AGA TGC T |  |
| sgVTN1-enh#1 | GG AGC ATA ACA GGC AGA GAG |  |
| sgVTN1-enh#2 | GT AAT GAG TTA GAA AGT GAT G |  |

**Table S3. List of antisense oligonucleotides (ASO) used in this study**

| ASO name | Sequence (5 🡪 3)  + indicates Affinity Plus (locked nucleic acid) base  * indicates phosphorothioate bond |
| --- | --- |
| EDN1e_ASO#1 | +A*+A*+A*A*G*A*A*A*G*A*G*T*T*+C*+T*+G |
| EDN1e_ASO#2 | +T*+A*+T* T*C*T*G*A*T*T*C*A*G*+A*+C*+A |
| EDN1e_ASO#3 | +T*+C*+T*G*A*A*A*C*A* A*A*G* G*+C*+T*+T |
| EDN1e_ASO#4 | +A*+A*+T*G*A*G* A*T*A* A*C*C* T*+A*+C*+C |
| Control_ASO | +A*+C*C*A*T*G*T*G*A*T*C*G*+C*+G |
